# Receptor-Assisted Nanotherapeutics for Overcoming the Blood–Brain Barrier

**DOI:** 10.1007/s12035-024-04015-9

**Published:** 2024-04-01

**Authors:** Akshada Mhaske, Shalini Shukla, Kailash Ahirwar, Kamalinder K. Singh, Rahul Shukla

**Affiliations:** 1 Department of Pharmaceutics, National Institute of Pharmaceutical Education and Research (NIPER)-Raebareli, Bijnor-Sisendi Road, Sarojini Nagar, Lucknow, Uttar Pradesh 226002 India; 2 School of Pharmacy and Biomedical Sciences, University of Central Lancashire, Preston, PR1 2HE UK; 3 Biomedical Evidence-based Transdisciplinary Health Research Institute, University of Central Lancashire, Preston, PR1 2HE UK

**Keywords:** Blood–brain barrier, Receptors, Transporters, Nanotechnology, Nanomedicine, BBB disruption

## Abstract

Blood–brain barrier (BBB) is a distinguishing checkpoint that segregates peripheral organs from neural compartment. It protects the central nervous system from harmful ambush of antigens and pathogens. Owing to such explicit selectivity, the BBB hinders passage of various neuroprotective drug molecules that escalates into poor attainability of neuroprotective agents towards the brain. However, few molecules can surpass the BBB and gain access in the brain parenchyma by exploiting surface transporters and receptors. For successful development of brain-targeted therapy, understanding of BBB transporters and receptors is crucial. This review focuses on the transporter and receptor–based mechanistic pathway that can be manoeuvred for better comprehension of reciprocity of receptors and nanotechnological vehicle delivery. Nanotechnology has emerged as one of the expedient noninvasive approaches for brain targeting via manipulating the hurdle of the BBB. Various nanovehicles are being reported for brain-targeted delivery such as nanoparticles, nanocrystals, nanoemulsion, nanolipid carriers, liposomes and other nanovesicles. Nanotechnology-aided brain targeting can be a strategic approach to circumvent the BBB without altering the inherent nature of the BBB.

## Introduction

A meticulous transmission between neuronal networks demands the segregation of the central nervous system (CNS) from peripheral organs that is attained via the blood–brain barrier (BBB). It is a highly distinguishing structure enriched with specialized cells that coordinates transmission of molecules across neuronal and peripheral compartments. However, the BBB restricts various neuroprotective biomolecules and limits the overall transportation of therapeutic molecules in the CNS. Different transportation schemes are being exploited for the direct bypassing of the BBB via noninvasive and invasive approaches. Hence, for successful transportation of drug molecules across the BBB, medicinal, physicochemical and pharmaceutical approaches need to be taken into consideration. Improvement in physicochemical properties of drug for BBB-mediated transportation via noninvasive strategy is one of pharmaceutical approach. Since past few years, nanotechnological approaches are being utilized for improvement in drug targeting efficiency to overcome the obstacles in CNS delivery [1]. Nanotechnology is a reliable platform for delivery of drug molecule irrespective of its physicochemical characteristics, as these nano-entities encapsulate drug molecules with inherent hydrophilicity and lipophilicity. Although various carriers have been exploited for delivery of neurotherapeutics, target-orientated nanotherapeutic delivery is more precise for brain targeting. As the BBB is encompassed with various receptors, transporters and channels hence, drug-enclosed nanocarriers uptake via these routes can improve the extent of drug transport. Further understanding of the BBB’s inherent nature and formulation strategies to utilize these pathways without damaging the BBB is a prerequisite for development of successful neurotherapeutics. In this review article, we have discussed the structural orientation of the BBB, therapeutic delivery strategies across the BBB, interplay between neurological sequelae and BBB integrity, factors influencing BBB destruction and nanotechnological façade for delivery of neurotherapeutics [2, 3].

## Fundamentals of the Blood–Brain Barrier

For the maintenance of homeostatic conditions of neurons, a continuous availability of basic nutrients and oxygen-rich environment is a prerequisite condition. Conservation of CNS homeostasis is essentially critical, as neuronal cells are highly sensitive towards various xenobiotics and slight exposure to such entities can disrupt the overall balance of the CNS. For providing protection against such challenges, brain capillaries and vascular network are embedded into the BBB layer. The BBB is an exclusive multilayer being a biochemical and structural barrier that keeps fulfilling the need for nutritional and oxygen consumption for the well-being of intracerebral environment, thereby ensuring the protection of cerebral milieu from xenobiotics and environmental factors.

### Structural Orientation of the Blood–Brain Barrier

Morphologically, the brain vascular system is like peripheral blood transporter network, where brain capillaries are attached to the marginal ends with the brain parenchymal cells. The BBB comprises blood capillaries arranged with other specialized cells such as tight intracellular junction of endothelial cells, astrocytic cells, pericyte network and neuronal appendages. Astrocytic cells are situated above the basal laminal layer that gives rise to strong prohibitory barrier. Pericyte network composed of unit pericytes being a subtype of mesenchymal cell settles within the perivascular gaps between capillaries and astrocytic feet-like projections. To attain the selectively restrictive permeation ability, endothelial cells show specific characteristic arrangement compared to peripheral endothelial cells which resulted in (a) reduction in intracellular flux, (b) intracellular tight junctions impose the charge-based resistance at paracellular gap that restrict the flux, (c) endothelial cells direct a specialized transportation mechanics and (d) higher mitochondrial count present at endothelial junction facilitates higher metabolic rate at junction. At the cellular level, adherens junction (AJ) and tight junctions (TJ) supervise the structural integrity present in the BBB. Tight junctions are formed between cerebral-originated endothelial cells and choroid plexus derived from epithelial cellular structures. TJs also recognized as zonulae occludentes provide the significant protection to the CNS via limiting the permeation extent of hydrophilic molecules via diffusion-based paracellular pathway from peripheral blood circulation [4]. AJ is composed of nectin and cadherin (proteinoid) adhesions which stretch between cell cytoplasm and intracellular entities via catenin scaffolding–based α,β,γ-catenin and forms a sturdy and strong framework for the overall structural integrity of the BBB. The TJs are surrounded with occludin and claudin intracellular protein and junctional adhesive molecules (JAMs). Occludin and claudin are attached with cytoplasmic extensions, with 20 different isoforms of claudins reported for involvement in TJ structure. Under various experimental studies, loss of claudin isoforms is reported in glioblastoma and encephalitis. This loss is correlated with the disruption of BBB coherence due to disease conditions. In terms of cerebral encephalitic conditions, altered BBB permeation ability is reported. Severity of disease is interlinked with the extent of BBB disruption. In genetically altered mice, the absence of claudin-5 isoform is more likely to have leaky and compromised BBB and ends up in premature death. The TJs are known to impose severe impediment in paracellular-based diffusion kinetics amongst endothelial cells and hydrophilic molecules. Such alteration in paracellular diffusion is imposed by TJ due to high resistance of the BBB nearly more than 1500 Ω/cm^2^. Such low conductance rate of paracellular diffusion reveals impact of tight intracellular junction ability to maintain the integrity of BBB structure [5, 6].

## Development of the Blood–Brain Barrier

**Fig. 1:**
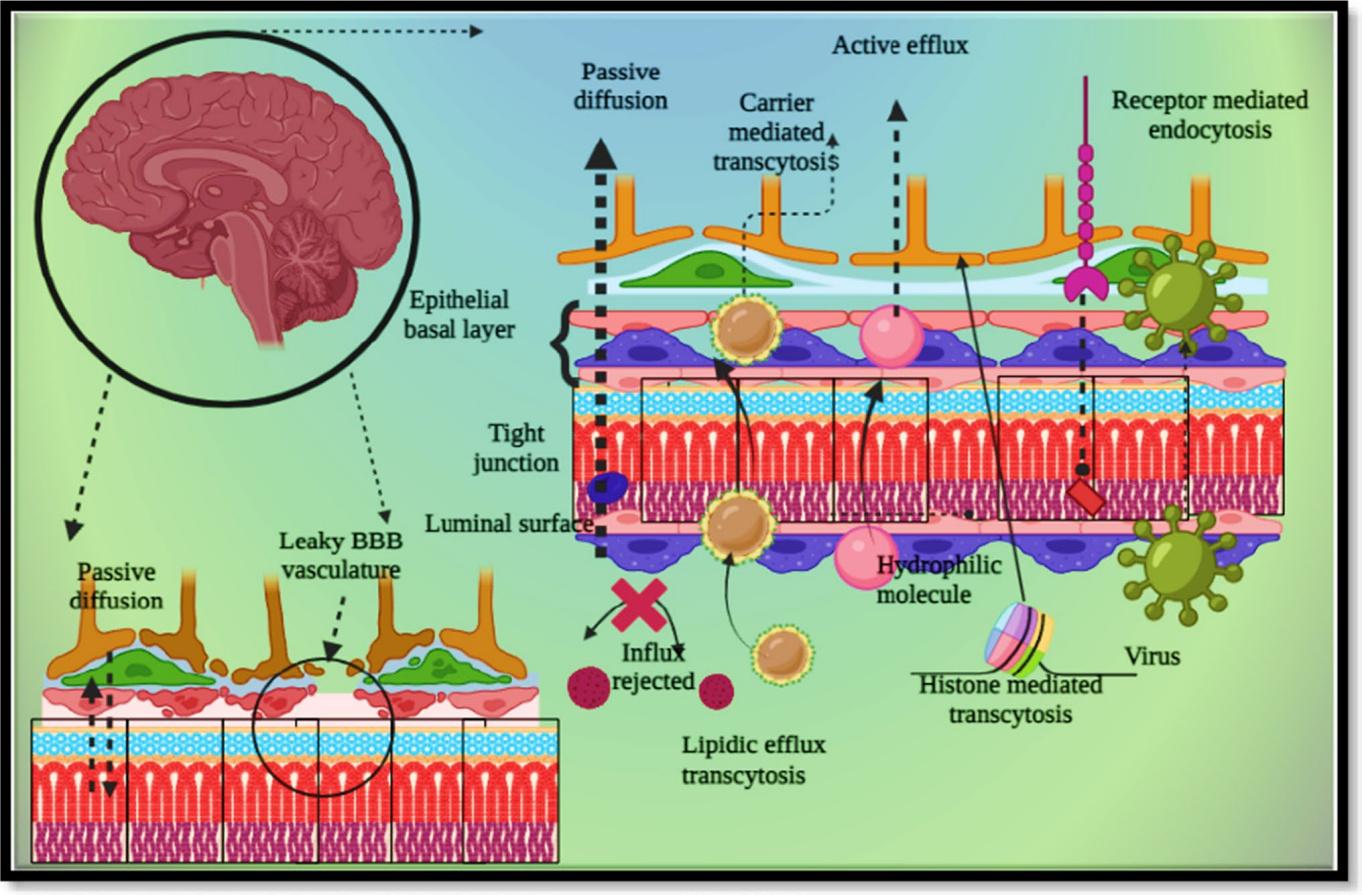
Structural representation of the blood–brain barrier and its interface. The BBB is a prime checkpoint which selectively allows permeation of essential molecules. Biomacromolecules can be transported across the BBB via various pathways such as receptor-mediated trans-endocytosis, active targeting, passive diffusion and few other allied mechanisms. The brain microvasculature interface is made of endothelial cells, astrocytic projection and pericyte network

The BBB assembly furnishes via angiogenesis pathway, and process begins when primary vessels differentiate into neuronal ectoderm during embryonic phase, and acts as a pre-existing blood vessel for future new vessels. These pre-existing vessels give rise to TJ and nutrient carrier channel. Upon maturation, these vessels develop and act as a primary vesicle that meets astrocytes and pericytes. Such maturation can efficiently decrease transcytosis extent, enhance efflux expression and reduce the leucocytic affinity. Maturation can be simplified as sealing of adjacent endothelial TJ and mainly seen after completion of embryonic development. The BBB is originated from the perivascular neural plexus covered with neuronal appendages, and such foundation assembles the complex process of central nervous system development. Angiogenesis is the key pathway for developing perivascular neural tissues within mesenchymal neural region, which allows BBB establishment and blood capillary alignment and development within primitive encephalon. Nutritional circulation within brain capillaries associates with neural homeostasis via transmission of progenitor cells within brain compartment [7]. Besides the inherent protective nature of the BBB, it is also modulated for the delivery of antibodies, peptides, proteins, albumin and various essential micromolecules for maintenance of neural well-being. Transportation of micromolecules is a resultant of conjoint efforts of cerebral nutritional pool and diffusional pathways such as paracellular, transcellular and intracellular diffusional kinetics. Such diffusion can be linked with carrier-based uptake, receptor-kindled uptake, cell-facilitated uptake and adsorption-induced assimilation. The paracellular transcytosis is transmission of solubilized micromolecules amongst two distally adjoint tight junctional-endothelial junctions based on reverse osmosis gradient. Usually, smaller water-soluble fragments can easily traverse across paracellular pathways. Paracellular transportation may render enhanced permeability for other substantial or unwanted substances. Transcellular transportation is transmission of molecules via tight endothelial lining which is viable for lipidic molecules, light gases, ethanolic beverages and anaesthetics medicines. Ideally, lipophilic molecules radially cross the BBB and later mixed within the lipophilic matrices of vascular endothelial cells. Other than the BBB, various barriers are also involved to protect the brain from hazardous elements. Such protection is offered by an efflux transporter that sticks to the micromolecules and directs them back to systemic blood circulation away from central nervous compartment. For successful access within cerebral compartment, a molecule must surpass the BBB. Glucose and amino acids are quintessential elements for energy and protein synthesis that enter via active transport mechanism. To make such transportation at ease, various carriers or transporters get involved within cellular uptake within brain compartments. Adsorptive-mediated uptake is a process of traversing micromolecules and ligand-specific nanocarriers via the BBB. Adsorptive-based uptake is dependent on electrostatic reaction amongst anionic micromolecules and cationic receptors present on endothelial junctions. Although this procedure is nonselective in nature, that may be a resultant of drug deposition within other off-target organs. Under treatment strategies, delivering drug candidate within neural compartment is challenging and is dependent upon leucocytic transmission. Drugs with inherent lipophilic quotient or lipophilic carrier can attain access within brain capillaries easily via leucocytic assistance mainly via diapedesis that allow movement of drug within central neural compartment. Structural representation of the BBB is shown in Fig. 1 [8].

## Molecular Comprehension of BBB Transportation Voyages

The brain-targeted delivery primarily encounters various transportation kinetics receptors with ion-based channels adenosine triphosphate (ATP)-binding cassette (ABC) transporters and solute carrier (SLC) transporters, the two basic transporters specific for different types of molecule.

### ABC Transporter

ABC transporters are commonly localised within vascular endothelial cells and, to a lesser extent, expressed in astrocytic, neuronal and microglial projections. ABC transporter is the largest protein superfamily commonly expressed in various living organisms ranging from bacteria to human. These transporters regulate different essential activities like nutritional assimilation, cell signalling pathway and conveyance of peptide, ions and substrates. Additionally, these synchronize the transportation of toxic substrates across different physiological barriers to preserve physiological homeostasis. Transporters mainly modulate the bioavailability of CNS drug molecules by simulating uptake, distribution profile and excretion kinetics. Thereby, ABC transporter is a crucial defence system of the BBB. The ABC protein molecules being an active transporter efflux the substrates in a concentration-dependent manner, and these transporters have a driving force derived from hydrolysis of adenosine-5′-triphosphate which thus maintains the intracellular amount. Amongst the various transporters, ABC family transporter is subdivided into 49 members of superfamily. This superfamily is classified in 7 subtypes, viz. ABC_A_ (12 types), ABC_B_ (11 types), ABC_C_ (13 types), ABC_D_ (4 types), ABC_E_ (1 type), ABC_F_ (3 types) and ABC_G_ (5 types). These subtypes catalyse the transportation mechanism for steroids, lipid constituents, xenobiotic agents, peptide fragments and antigen antibiotics. Amongst 49 subtypes of ABC transporter, 29 different ABC transporter subtypes are expressed within neuronal compartment. ABCB1 or permeability glycoprotein 1 (P-gp) is a long glycoprotein domain of the multidrug resistance protein 1 (MDR-1) present on the endothelial lining on the BBB, cerebral parenchyma and glial and neuronal cells. P-gp is a ubiquities homologous transmembrane protein within plasma membrane. Strategic expression of P-gp on the BBB luminal wall enhances shielding of neural compartment against xenobiotic agents, endogenous peptides, ligands and neuroprotective drugs. Hence, P-gp is recognized as a prime ABC transporter that performs the function of CNS protection and drug resistance. P-gp efficiently restricts the ingress of xenobiotics into neural compartment, while this feature also curbs the access of neuroprotective agents. Such hindrance lowers the extent of neuroprotective moieties’ entrance via the BBB. ABC efflux transporters have broad-spectrum substrate selectivity, and various hormones, cytokines, phospholipids, sphingolipids, prostaglandins, amyloid-β and aldosterone are P-gp substrate molecules [9]. ABCC family or multidrug resistance–associated protein (MRP)-1 is another pivotal subfamily of ABC transporter family. MRP is a marker for the blood-cerebrospinal fluid (CSF) barriers, as it is aggravated on a basal layer of choroid plexus–originated epithelia. MRP-specific substrates are sulfate-conjugated species, glutathione, glutathione-associated leukotrienes, glucuronide-associated proteins and prostaglandins. Various neuroprotective drugs are reported as P-gp and MRP substrates and this transporter-based resistance has been associated with their poor treatment efficiency. Considering the hindrance of transporter for successful delivery of neurotherapeutic agents, few reciprocating strategies such as (i) P-gp inhibition by usage of P-gp inhibitor drugs, (ii) by employing the structural activity of relationship for development of neuroprotective drug which conserves the essential pharmacological impact without being detected by P-gp transporter and (iii) incorporation of drug within carrier vector via Trojan horse mechanism to bypass the efflux transportation by ABC transporter. Few P-gp inhibitors include verapamil, quinidine, cyclosporin, colchicine, digoxin, erythromycin and many more, whereas sulfinpyrazone, probenecid and benzbromarone are analysed for their multidrug resistance protein inhibition ability. Second approach for keeping the ABC reactivity out of drugs includes the knowledge of structural activity relationship. This approach emphasizes on the essential chemical moieties such as less hydrogen bond donor species, less positive energy, high lipophilicity and low polar species. The characteristics like high hydrogen acceptor bonds, higher lipophilicity, presence of more than one aromatic ring and higher molecular weight structure increase the probability of drug as a P-gp substrate. And the last approach is utilization of a suitable peptide-based vector for bypassing the efflux transporter. Mechanistically hydrophobic chains of peptide gain entry in the plasma membrane, and negatively charged species reacts with the positively charged tails of peptide domain. This vector-based delivery is based on absorptive-endocytosis mechanism [10].

### Solute Carrier Transporter (SLC Transporter)

SLC transporter is the second most important drug transport protein at endothelial BBB junction. In opposition to ABC transporters, SLC membrane transporter is facilitated by gradient electrochemical manner by utilization of organic and inorganic salts. SLC transporter is broadly subdivided into 65 superfamilies. Out of which, 43 different types of SLC transporter are detected in a human body, and SLCO, SLCO-22 and SLCO-21A subtypes are present at the neuronal BBB junction. SLC transporter mainly allows transportation via facilitated diffusion mechanism. SLC2A1 supports the glucose transport across the BBB junction on the expenditure of pre-existing ion concentration in unidirectional ionic exchange, whereas SLC2 (glucose transporter (GLUT)), SLC3 (amino acid transporter), SLC4, SLC5 and SLC6 perform the functioning of bicarbonate and Na^+^-Cl^−^-dependent neurotransmitter transporters. The SLC functionalization is largely affected by the astrocytic manipulation. Prime SLC subfamilies have firm emphasis on the targeting efficiency of neuroprotective drug for the treatment of neurological challenges [11]. For instance, SLC22A is highly expressed in astrocytic projections within various neuronal regions such as hippocampus, nigrostriatal axis and hypothalamus loci and is a competent transporter for the neurotransmitters like epinephrine, norepinephrine and histamine. The SLC22A subfamily is reported for its application in improving targeting efficiency of psychotropic agents. The SLC1A2 and SLC1A3 are Na^+^-dependent glutaminergic transporters exploited well in targeting neurological challenges including epilepsy, Parkinson’s disease, depression, Alzheimer’s disease and amyotrophic lateral sclerosis. SLC17A and SLC32A family is mainly present within neuronal structure and thereby involved in transportation of glutamine and gamma-aminobutyric acid. Brain synchronizes both peripheral and neuronal coordination that escalates in higher glucose and oxygen demand which are obligatory metabolic substrates for regular brain function. As glucose is a hydrophilic molecule mainly transported via a SLC2A transporter, ten different SLC2A subfamilies are involved in brain glucose transportation. Mutational alteration within SLC2A transporters is in plausible linkage with development of intellectual affliction, ataxia (rare disease associated with glucose insufficiency) and epileptic episodes. SLC transporters superintend the metabolite compounds and nutritional in and out transportation flux to maintain the cerebral homeostasis, neurotransmission and clearance of metabolic waste. A total 80% of total imported glucose is utilized in GABA-glutamate cycle although brain requires 20% of total body glucose for smooth functioning. As SLC17A and SLC32A are involved in glutaminergic and GABAergic vesicular-mediated transport towards presynaptic terminal. Successful synaptic channelling needs the sustained neurotransmitter release from synaptic vesicle channels. Dissociated neurotransmitters bundle travel through synaptic cleft, where released neurotransmitters affix on the specific receptors and later excrete from synaptic cleft by glial uptake. During this whole neurotransmitter release and uptake process, packaging of neurotransmitters is a crucial first step regulated by SLC18A2-3, and this transporter assembles the bundles of histamine, norepinephrine, serotonin, epinephrine and acetylcholine at synaptic junction. Most commonly SLC17A6-7 (glutaminergic bundle), SLC32A (GABAergic bundle), SLC5A7 (packs acetylcholine bundles) and SLC6A transporters are involved with GABA, serotonin and dopamine bundle packaging [12].

## Blood–Brain Barrier Crossing Strategies and Challenges

For traversing the BBB, various pathways and strategies have been employed such as receptor-specific delivery, ligand-targeted delivery and colloidal-based delivery. All the reported strategies are discussed in this section.

### Transcytosis

Transportation across the brain and peripheral vascular system is controlled by endothelial cells situated on BBB junction. Endothelial cells present on the BBB allow transfer of protein and nutrients from peripheral blood to the CNS and vice versa for removal of toxic byproducts from central compartment. Endothelial cells at the BBB junction are highly specialized cells as these cells are chiefly involved in maintaining the overall integrity of central neural compartment. Endothelial cells can easily allow access to the high molecular weight proteins or nutrients via endocytosis pathway. The BBB junctional cells via exosome mediated fusion with peripheral plasma membrane, high molecular protein compounds released across epithelial junction via endocytic secretory pathway. Under this pathway, gradual pH alteration within endosomal compartments shifts to 6.5–5.5 range and later lysosomal pH shifts to 4.5 due to ATPase proton pump mechanism. This pH shifts can facilitate lysosomal protein breakdown into smaller fractions. To tackle such lysosomal degradation, selection of appropriate receptor for receptor-based uptake of peripheral proteins via secretory mechanism is necessary. Nonetheless, carriers that dodge the endosomal acidic pH conditions can successfully gain entry within brain via passive diffusion mechanism [13, 14]. Although with all the understanding, an exact mechanism harbouring the cargoes across the BBB junction is still plausible. Furthermore, few probable mechanisms put forth are as follows: (i) clathrin-based, (ii) caveolae-based, (iii) caveolae-clathrin-based and (iv) micropinocytosis. Unknown transporting trajectories and mechanism can be understood by means of covalent dye–based conjugation of cargo molecule. A slight change in arbitrary compound can deviate transportation mechanics amongst label-free cargo and labelled cargo molecules due to involvement of various trafficking mechanisms. Various experiments also confirmed the modification of endocytosis mechanism via a linker of photosensitizing agent [15].

### Receptors Mediated Blood–Brain Barrier Delivery

The basic strategy introduced in the late 1990s for effective transport of biological neuroprotectants in the CNS utilized surface adsorption or conjugation of receptor-specific moiety with remedial interest. The biological moiety can be either peptide-based ligand, receptor-specific antibody or endogenous receptor ligand. Such receptor-mediated biologics transportation approach has been applied for delivery of RNA or DNA fragments, monoclonal antibody mimicking protein, recombinant bioactive and nanomedicines incorporating neuroprotective agents. The receptor-mediated targeting applied primary adaptation by tethering the targeting moiety and neuroprotective entity via chemical linkages and, secondly, amalgamation of nanovesicle and receptor targeting moiety for the anticipated therapeutic interest. Although receptor-mediated targeting was introduced in neurological field at least two decades ago, its application has increased in recent years owing to pharmaceutical and academic efforts [16]. Various receptors that have been explored for such delivery are listed as follows.

#### Nicotinic Acetylcholine Receptors

**Fig. 2:**
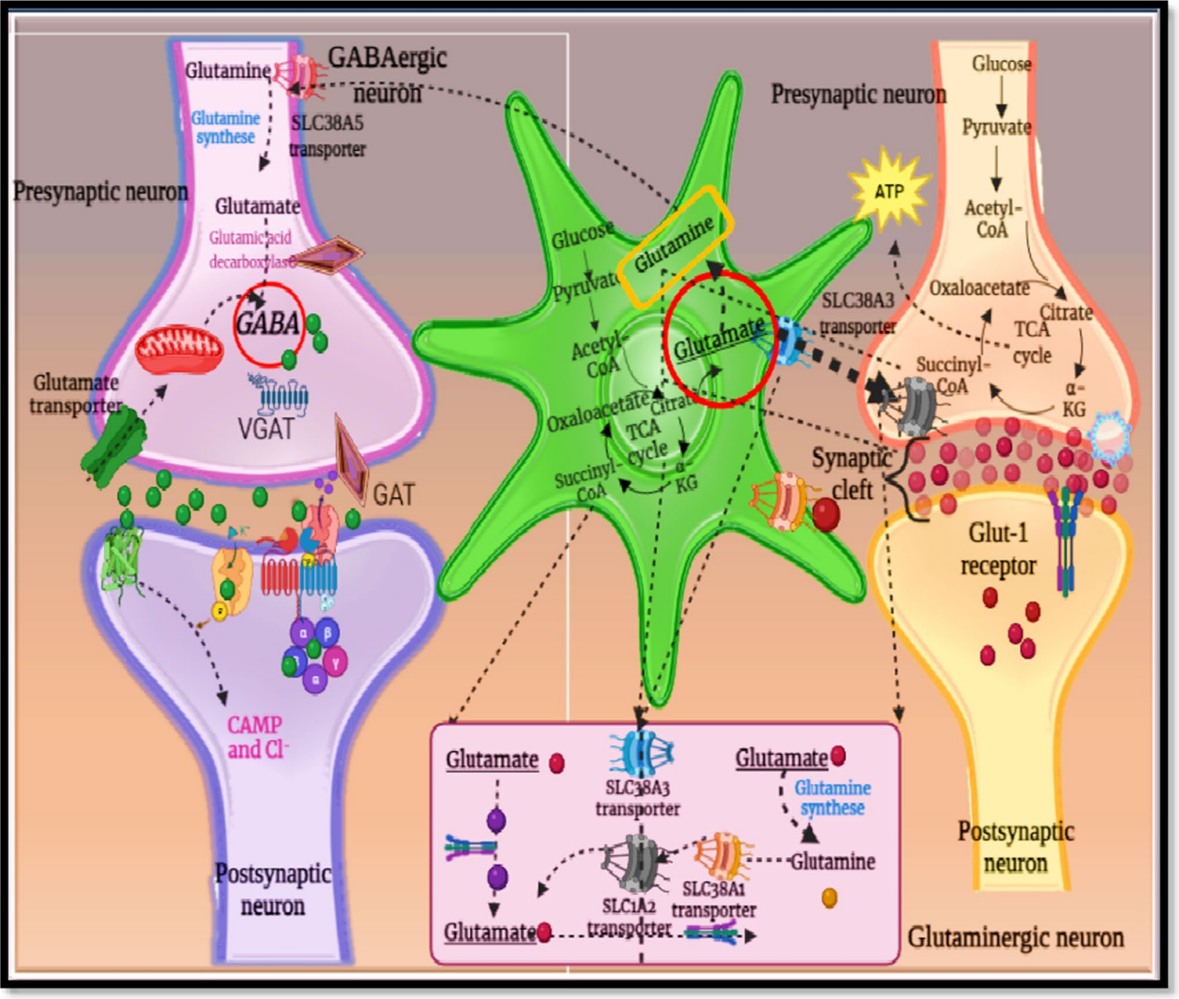
Nicotinic acid cycle. Nicotinic acid receptor is as an essential receptor for successful uptake and utilization of GABA and glutamate. Maintenance of these two neurotransmitters within neuronal network is quintessential as dyshomeostasis within these two neurotransmitters can lead to development of neurological challenges

Nicotinic acid receptor (nAChR) is a heterogeneous ion channel receptor in the nervous system, and these receptors are specific for endogenous neurotransmitters such as nicotine and acetylcholine. The nAChRs are widely exploited for substance abuse and thoroughly studied for drug aftereffects in clinical analytical experiments. Generally, nAChRs are recognized for their impact on the physiological and psychological level and maintenance of the cerebral homeostasis. However, reported alteration in the nicotinic cholinergic receptor-mediated signal conduction may be linked with cerebral development and neurological challenges along with neurotransmitter deactivation. The nAChR group forms a heterogenic pentamer orientation of oligomeric strand within different nervous compartments. Being a part of Cys-loop superfamily, nAChRs have homologue orientation like other subfamily receptors such as muscarinic AChR, GABA, serotonin (5-HT receptor) and glycine receptors. These receptors are available in different subtypes based on the composition of encoded genes. The nAChRs are structurally arranged in such a fashion that pentameric subunits assemble around central pore channel located in synaptic cleft, with extracellular hydrophilic terminal known as ACh binding site. Reportedly, nicotinic receptor can transport rabies virus glycoprotein (RVG) protein within predetermined cell of interest. Recent upgradation of RVG protein in RVG29 is widely exploited in pharmaceutical and academic research purposes. RVG29 is a 29-membered amino acid chain isolated from rabies virus glycoprotein. Recently, leptin 30 (a 30-amino acid-containing leptin peptide) is implied with dendrimer-based nanovesicles with dendrigraft poly-l-lysine (gene vector). Brain capillary endothelial cells (possess leptin receptor) showed enhanced uptake of leptin 30–tagged nanovesicles, and in vitro BBB model reveals higher transfection capability to traverse the BBB [17]. Pinheiro and coworkers [18] have conjugated quercetin-loaded solid lipid nanoparticles (SLNs) and nanolipid carriers (NLCs) with RVG29 peptide for improved brain efficiency in Alzheimer’s disease. The peptide conjugation was confirmed with FTIR analysis, and further hCMEC/D3 cell line studies exhibited no cytotoxic behaviour of prepared formulation, while peptide conjugation enhanced permeability in a BBB model. Further thioflavin T assay revealed better ability as a neuroprotectant against amyloid beta (Aβ) fibrillation [18]. The functioning within nicotinic receptor is shown in Fig. 2. DCDX, a D-peptide ligand for nAChRs, enhances drug delivery to the brain via liposomes. Han and colleagues [19] evaluated mechanistic insights of DCDX-modified liposomes in the BBB via in vitro and in vivo analysis. Predominant transport occurred through the lipid raft/caveolae endocytic pathway, involving the endoplasmic reticulum (ER) and Golgi complex. DCDX-modified liposomes also engaged the endosome/lysosome pathway. Notably, nAChR α7 did not influence their transportation. P-gp emerged as the primary efflux transporter, and inhibiting P-gp enhanced therapeutic efficacy against glioblastoma in a mouse model. These findings highlight DCDX-modified liposomes as a potential tool for glioma treatment [19]. In an another study, the specific neural cell targeting capabilities of rabies virus derived peptide (RDP) via the nAChR have been demonstrated as promising approach to the delivery of therapeutics to the brain [20].

#### Glucose Receptors

**Fig. 3:**
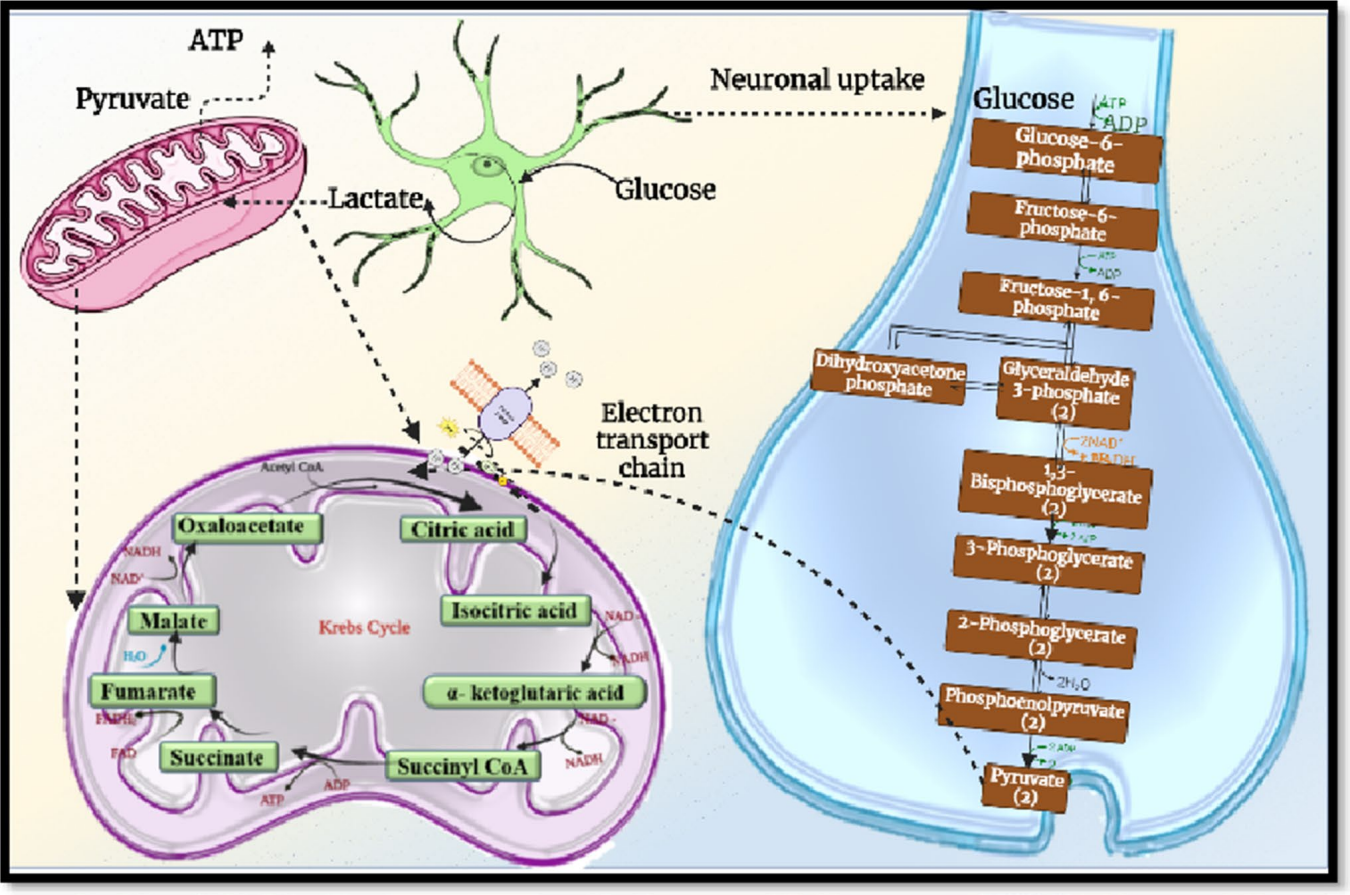
Glucose utilization within glucose transporter and neuron. Glucose is essential for maintaining homeostasis within the brain and in energy production of peripheral organs. Glucose mainly uptake via electron transport chain further undergoes glycolysis process within neuron and forms pyruvate molecules which further utilized via mitochondria and generates ATP molecules

More than 20% of administered glucose is assimilated by brain itself, as brain requires energy for catalysation of biogenic reactions. To gain access within brain compartment via bypassing the BBB, a molecule should possess sufficient lipophilic and hydrophilic balance, and as glucose is evidently hydrophilic, its uptake via the BBB is hindered. Hence, a dedicated transporting portal or chaperone system is essential for glucose uptake via the BBB. Brain cells are fortified with glucose receptor for selective uptake of glucose. Shortly, once glucose molecules reach within extracellular brain spaces, it is quickly assimilated by GLUT receptors and supplied within neural compartment. The glucose receptors in brain are a subtype of SLC2 superfamily comprising SGLT-based Na^+^ transporter specific for glucose. GLUT is subdivided into GLUT1, GLUT2, GLUT3 and GLUT4 according to functional modification and site of availability. GLUT1 is present at brain stem, placental cells and erythrocytes. GLUT2 is located at liver, pancreas and kidney cells. GLUT3 is present within neurons and placenta, whereas GLUT4 is found in adipose tissues, heart and other peripheral muscles. GLUT1 is a hydrophobic transporter with 12 pairs of spanning membrane α-helix, and each helix is encoded with 20 amino acids. These α-helixes are amphipathic in nature comprising one end with polar appendage, and the other is nonpolar; such behaviour allows collection of few such transporters that lead to the formation of glucose-specific pore channel that specifically allows glucose molecule via a gateway towards brain compartment. The glucose uptake via GLUT receptors is mainly dependent on concentration gradient. This concentration gradient is formed between brain interstitial space and blood compartment, and such upsurge of glucose can be seen after meal consumption. Once glucose in entrapped via GLUT1 receptor, it is taken up via various organelles. In cerebral compartment, assimilated glucose is further broke down via glycolysis and utilized for other metabolic reactions as shown in Fig. 3 [21].

Contemporary therapies involving antisense oligonucleotides (ASOs) for the management of CNS disorders often necessitate invasive administration methods, imposing a substantial burden on patients. To mitigate this challenge, Min et al. [22] developed a novel approach for the systemic delivery of ASOs to the brain by traversing the BBB through glycaemic control as an external trigger. In this context, polymeric nanocarriers equipped with glucose moieties have been devised and designed to bind specifically to GLUT1 expressed on brain capillary endothelial cells. These nanocarriers, featuring a particle size of approximately 45 nm and an optimized glucose ligand density, facilitate the stable encapsulation of ASOs. Following intravenous administration, the optimized nanocarrier efficiently accumulates in brain tissue within 1 h, demonstrating significant gene knockdown for a target long noncoding RNA across various brain regions, including the cerebral cortex and hippocampus. These findings underscore the efficacy of glucose-installed polymeric nanocarriers, offering a noninvasive avenue for ASO administration to the brain. This approach holds promise for the treatment of CNS disorders, providing a more patient-friendly alternative to current invasive methodologies [22].

#### Transferrin Receptors

**Fig. 4:**
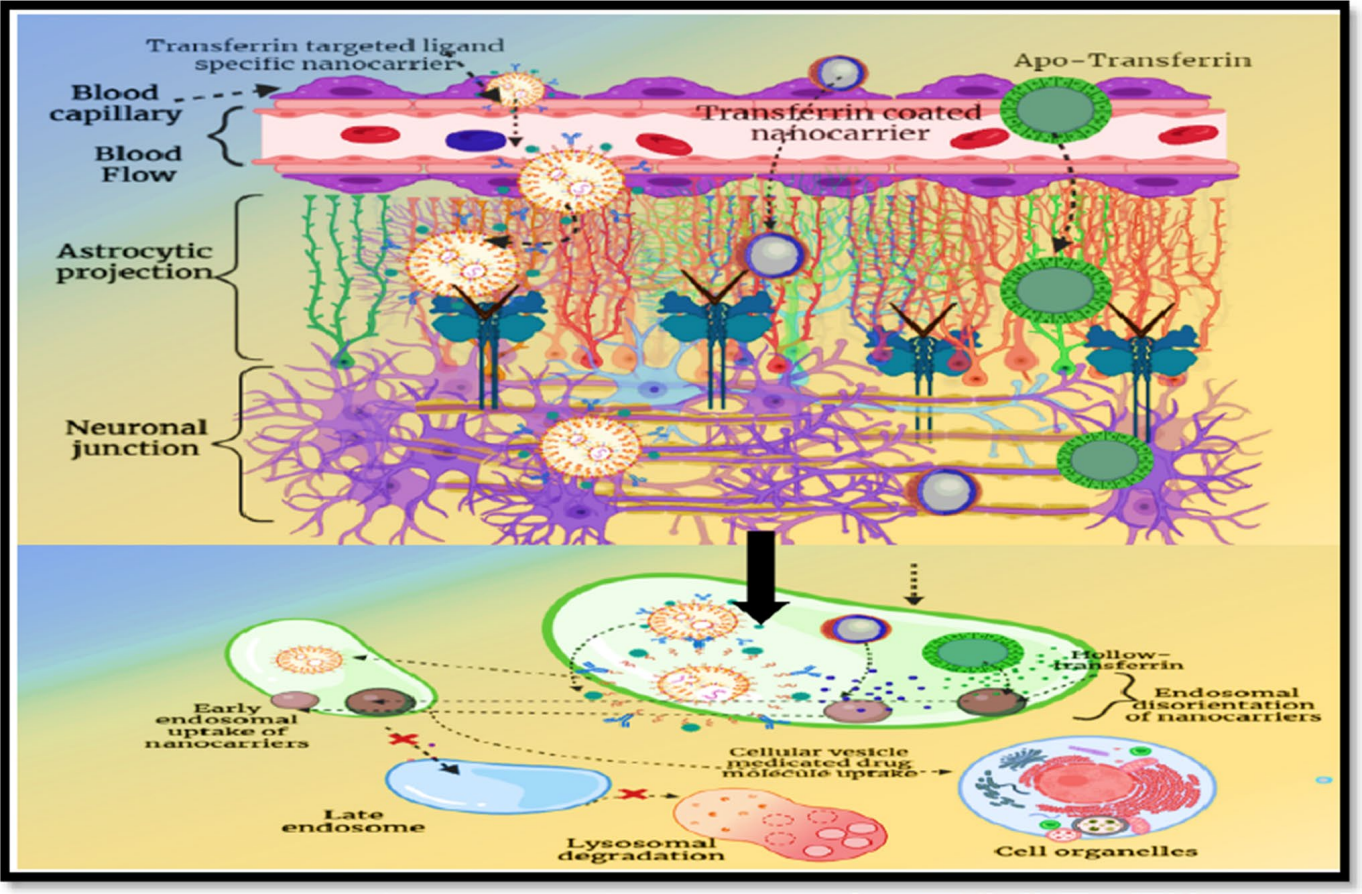
Transferrin-targeted drug transcytosis. Transferrin is a leading and most exploited receptor for strategic endocytosis of drug molecules. Ligand-conjugated or transferrin-coated nanocarriers assimilated via the receptor bridges and further uptake via early endosome and thereby release the drug content within cellular vesicle

For maintenance of cerebral homeostasis, ionic balance within brain compartment is essential. Compared to other metal ions, iron has gained special interest in cerebral biological interactions. Iron catalyses various important biological pathways like mitochondrial respiratory cycles, DNA development pathways, oxygen carrier kinetics, neurotransmitter release, storage and synthesis along with myelin formation within neuronal compartment. Owing to these exquisite features offered by iron, a special iron transporting receptor–based intervention is present for smooth uptake of iron within brain. Transferrin receptors (TfRs) are enriched with transferrin glycoprotein units, and these receptors are known for their iron transportation ability [23]. Transferrin (Tf) proteins are mainly formed within liver cells, BBB junction and mammary cells. Structurally, Tf consists of polypeptide chain with two different binding domains. Both the domains possess at least one site available for ferrous ion binding. Ferrous binding efficiency is higher at the 7.4 pH value, and at this pH environment, binding efficiency is at its peak that thermodynamically binds ions to these sites. In the peripheral and at the junctional bridges, ferrous ions are assembled in various forms with transferrin protein as a mono-ferric, di-ferric and apo-ferric (no ferrous ion) ionic assembly. Tf is specific for ferrous ions along with other metal ions such as aluminium, copper, manganese and cadmium. Although Tf can allow binding for other metal ions, ferrous binds to these sites more firmly and can potentially displace other metal ions. Tf protein allows ferrous transportation towards transferrin receptor via the receptor-facilitated endocytosis [24]. The transcytosis within Tf-targeted nanocarrier is depicted in Fig. 4. Jain et al. [25] investigated permeation of bioactive molecules across the BBB utilizing polysorbate 80–coated poly-lactic-*co*-glycolic acid (PLGA) nanoparticles (NPs) encapsulating methotrexate-transferrin (Tw-Mtx-Tf-NP) conjugates (Mtx-Tf). The facile trans-BBB migration of the engineered formulations through endocytosis, coupled with the inhibition of the P-gp efflux pump in the brain, was substantiated by the incorporation of Pluronic F-68 and/or polysorbate 80. The heightened expression of Tf receptors on the surface of cancer cells facilitated the targeted and sustained delivery of Mtx-Tf conjugates to brain cancer cells through receptor-mediated endocytosis. The developed formulations exhibited enhanced penetration relative to nontargeting experimental NP controls. To assess the transport potential and biodistribution of these nanosized polymeric carriers, which demonstrated successful migration and trans-BBB passage, FITC-labelled drug-loaded NPs were administered intravenously to albino rats. The antitumour efficacy of the newly formulated drug-loaded NPs was validated in comparison to controls using an experimentally induced tumour-harbouring rat model. The findings of the study indicate improved compatibility, reduced organ toxicity and enhanced antitumour activity of the developed formulations, attributable to their targeting and sustained delivery capabilities in cancer therapeutic interventions. In conclusion, studies substantiated both the targeted and sustained drug delivery potential of the NPs in vitro and in vivo. The formulated novel delivery vehicle demonstrates its utility in the advancement of tools for the treatment of brain cancer [25].

#### Leptin Receptors

Leptin (Lep) receptors are the subtype of cytokine I receptor with three prominent regions: intracellular compartment, extracellular region and transmembrane port. Lep receptors are known for their regulation ability at adipose tissues. Primarily, Lep receptors are also called as OB-R, and it is found in two isoforms, namely shorter OB-Ra and longer OB-Rb isoforms. OB-Ra is located mainly within endothelial brain capillary and thereby facilitates the leptin-based transcytosis process. In one such attempt, g21 leptin sequence is conjugated on the surface of poly-*co*-glycolide nanoparticles, and furthermore, these NPs were tagged with tetramethylrhodamine via avidin–biotin method. Prepared labelled NPs administered via intravenous administration to rats resulted in enhanced nanoparticle uptake within mouse brain parenchyma. This study suggested that Lep g21–conjugated NPs improve their brain barrier crossing ability via targeting leptin receptor–specific moiety [26].

Complex liposomes were meticulously constructed, employing 1,2-distearoyl-*sn*-glycero-3-phosphocholine, dihexadecyl phosphate (DHDP), cholesterol and 1-palmitoyl-2-oleoyl-*sn*-glycero-3-phosphate (PA) with the purpose of serving as adept drug carriers for resveratrol (RES) and epigallocatechin gallate (EGCG). The surface of these liposomes was adorned with Lep to facilitate BBB penetration and rescue degenerated dopaminergic neurons. The neuroprotective potential of RES and EGCG against neurotoxicity was investigated, utilizing an in vitro neurodegenerative model established with SH-SY5Y cells subjected to 1-methyl-4-phenylpyridinium (MPP +). Results revealed that an escalation in the mole percentage of DHDP and PA correlated with an increase in particle size and absolute zeta potential value, concurrently enhancing the entrapment efficiency of RES and EGCG. However, this augmentation coincided with a reduction in the release rate of RES and EGCG, as well as the grafting efficiency of Lep. Notably, Lep/RES-EGCG-PA liposomes exhibited a heightened ability to traverse the BBB compared to their non-modified counterparts. The incorporation of PA and Lep into liposomes not only augmented cell viability but also enhanced target efficiency. Immunofluorescence findings demonstrated that the conjugation of Lep with liposomes facilitated the docking of human brain microvascular endothelial cells (HBMECs) and SH-SY5Y cells via the Lep receptor, thereby enhancing their ability to permeate the BBB and facilitate cellular uptake. Further analysis through immunofluorescence and western blot techniques indicated that RES and EGCG encapsulated within liposomes contributed to neural defence by mitigating apoptosis-promoting proteins such as Bcl-2-associated X protein and α-synuclein. Simultaneously, there was an augmentation in the levels of the apoptosis inhibitor protein B cell lymphoma 2, tyrosine hydroxylase and the dopamine transporter. In conclusion, Lep-PA liposomes emerge as a highly promising delivery system for potential treatment of Parkinson’s disease, substantiated by their capacity to enhance BBB penetration, cellular uptake and neuroprotective effects against apoptosis-inducing factors [27].

#### Insulin Receptors

Insulin is primarily recognized for its exquisite ability to transport glucose and amino residues within intracellular spaces. Insulin synthesis in brain is less evident, but expression of insulin-specific mRNA and receptor intervention is reported. These receptors are localised within hypothalamic region, hippocampal site, cerebral prefrontal cortex, amygdale and cerebellum. Insulin receptors (IRs) are assisted by GLUT8 and GLUT4 receptors in upcycling of glucose for further cellular metabolism [28]. IRs are involved in leptin, oestrogen, growth factors and amino acids like tryptophan and mediate dopamine and serotonergic pathways. Intracerebral insulin is important for synaptic nerve conduction, neuronal homeostasis, dendritic survival, memory and cognition [29].

Under one such study, carmustine (BCNU)-loaded SLNs were functionalized with the 83–14 monoclonal antibody (MAb) (83–14 MAb/BCNU-SLNs) for targeted brain delivery. HBMECs incubated with 83–14 MAb/BCNU-SLNs were subjected to staining to illustrate the interaction between the nanocarriers and expressed IRs. Additionally, the presence of poloxamer 407 on 83–14 MAb/BCNU-SLNs induced cytotoxicity in RAW264.7 cells and impeded phagocytosis by these cells. An incremental rise in the weight percentage of DYN, ranging from 0 to 67%, marginally diminished the viability of RAW264.7 cells while concurrently promoting phagocytosis. Furthermore, the in vitro transport capability of 83–14 MAb/BCNU-SLNs across the BBB was enhanced with an increasing weight percentage of Tween 80. The inclusion of 83–14 MAb on MAb/BCNU-SLNs stimulated endocytosis by HBMECs via IRs, thereby augmenting the permeability of BCNU across the BBB. In conclusion, the developed 83–14 MAb/BCNU-SLNs represent a promising drug delivery system for BCNU, demonstrating potential efficacy in the targeted transportation of this antitumour agent to the brain [30].

#### Brambell Receptor

Immunoglobulins are the intrinsic molecules, usually with a limited accessibility towards the BBB owing to their higher molecular weight. Such inherent characteristics exclude antibody-mediated transportation in the CNS. Nevertheless, B lymphocytes synthesized immunoglobulin G (IgG) antibodies radically gain access within neuronal compartment via lymphocyte uptake mechanisms. Brambell receptors or fragment crystallizable (Fc) regions are mainly divided into four different subtypes, namely FcR1, FcrR2, FcR3 and neonatal Fc receptors. The Fc receptor family is versatile, earlier recognized as an IgG transporter in connective placenta from maternal to foetal IgG distribution. Further studies have highlighted additional features of Brambell receptor such as albumin circulation, IgG transportation across the BBB, permeation associator and suitable transporter for different viruses and antigens. The chief function of Fc receptor is to extract IgG from neuronal compartment and refurbish it in the peripheral blood flow [31]. Autoantibodies are increasingly acknowledged for their pathogenic potential in a burgeoning array of neurological disorders. While myasthenia gravis stands as the quintessential antibody (Ab)-mediated neurological ailment, numerous conditions marked by Abs targeting neuronal or glial antigens have emerged in the last two decades. The efficacious therapeutic strategy in most of these conditions involves the depletion of humoral immune components, notably IgG, through interventions such as plasma exchange or immunoadsorption. The neonatal Fc receptor (FcRn), primarily expressed by endothelial and myeloid cells, plays a pivotal role in IgG recycling, thereby prolonging the half-life of IgG molecules. FcRn blockade serves to impede the binding of endogenous IgG to FcRn, compelling these antibodies into lysosomal degradation and resulting in IgG depletion. The augmentation of endogenous IgG degradation through FcRn-targeted therapies has proven to be a potent therapeutic approach in patients with generalized myasthenia gravis, and is presently undergoing clinical trials for various other neurological disorders, including autoimmune encephalopathies, neuromyelitis optica spectrum disorders and inflammatory neuropathies [32].

#### Low-Density Lipoprotein Receptor

The low-density lipoprotein (LDL) is a glycoprotein analogue belonging to lipoprotein receptor gene superfamily, mainly located in brain and liver cells. The site-specific distribution of LDL receptor–related protein (LRP) receptor is mainly located in macrophages, neurons, microglial cells and smooth vascular muscles. Being a trans-membrane-based receptor, LRP easily interconnects with proteinase enzymes, lipids, lipoprotein and specific ligands. LRP mainly catalyses chief homeostatic functions like Aβ clearance, lipidic glycoprotein metabolism, microglial transmission and synaptic nerve conduction. LRP is structurally divided into different units such as epidermal growth receptor–based cysteine-enriched repeats, ligand binding domain with cystine residual strands, transmembrane sites and cytoplasm containing tail along with extracellular β-propeller subunit [33]. Aβ peptide undergoes γ-secretase via precursor amyloid strands. Overaccumulation of these fragments is recognized as the most crucial cause for Alzheimer’s disease development and neurodegenerating pathologies. For withdrawal of Aβ deposition, LRP operates via apolipoprotein and macroglobulin or directly through LRP-situated ligand binding sites. Astrocyte is organelle associated with LRP, as it is involved in maintaining the BBB structure by secreting extracellular matrix. Matrix metalloproteinase is another zinc-based endopeptidase, with a capacity to degrade extracellular matrix–originated molecules. But, under physiological malfunction, metalloproteases digest extracellular matrix and disrupt BBB structural orientation. Various experimental evidences suggest the involvement of LRP in reduction of metalloproteinases via endocytosis engulfment. Free metalloproteases bind with the heparin proteoglycan sulfate prior to LRP-based internalisation. Angiopeps are an aprotinin-derived human-isolated protein mainly employed for LRP-targeted drug delivery. Brain metastases typically exhibit a predominantly multifocal, infiltrative growth pattern, often co-opting surrounding tissue, while maintaining the integrity of the BBB. Angiopep-2, known to bind to the LRP1 on brain microvascular endothelial cells (BMECs), facilitates transcytosis to traverse the BBB [34].

Beyond the role of tight junctions, it is increasingly recognized that low transcytosis significantly contributes to limiting BBB permeability. Under one such study, Guo et al. [35] reported on Angiopep-2-anchored NPs loaded with statins (S@A-NPs), designed to enhance LRP1 expression and overcome low transcytosis at the BBB. The findings demonstrate that S@A-NPs selectively elevate LRP1 expression on both BMECs and brain metastatic tumour cells, facilitating efficient and self-promoting penetration through the BBB. This occurs through Angiopep-2-mediated endocytosis and statin-induced upregulation of LRP1. Systemic administration of S@A-NPs loaded with doxorubicin (S@A-NPs/doxorubicin (DOX)) significantly extends the median survival of mice harbouring brain metastases. The notable efficacy in BBB penetration and specific targeting of brain metastases via angiopep-2-mediated mechanisms, coupled with the LRP1 upregulation induced by statins, positions S@A-NPs/DOX as a promising candidate for potential clinical management of brain metastases [35].

#### Diphtheria Toxin Receptors

Diphtheria receptor or heparin-specific epidermal growth factor is a glycoprotein transmembrane receptor, with transportation specificity for toxin. This receptor is specific as it is deprived of intrinsic ligand; hence, this receptor can be a suitable delivery portal as it nullifies the chances of competitive ligand binding. The toxin assimilation via this portal is mainly conducted by receptor-based endocytosis. Diphtheria receptor is mainly expressed at neurons, glial cells and endothelial junction, and overexpression of receptor is usually seen in various neurodegenerative diseases and brain tumour cases. Toxin is not an inherent substrate for the receptor as it is neurotoxic in nature. Therefore, nontoxic ligands can be internalised via caveolae-based mechanism and act as a shuttle-based substrate. Agarwal and colleagues [36] employed recombinant receptor-binding domains of diphtheria toxin (RDT) as a targeting ligand for NPs to achieve specific cellular homing. Diphtheria toxin is known to bind to heparin-binding epidermal growth factor-like growth factor (HB-EGF) via its receptor-binding domain, and HB-EGF is frequently overexpressed on the cell surface in various cancer types. Monodispersed, spherical PLGA NPs were synthesized and subsequently coated with RDT. Characterization of these RDT-coated NPs (RDT-NPs) was conducted using field emission scanning electron microscopy (FESEM) and FTIR spectroscopy. The flow cytometry and fluorescence spectroscopy demonstrated that the coating with RDT enhances the cellular uptake of PLGA NPs. Furthermore, investigation revealed that RDT-NPs are internalised through clathrin-dependent receptor-mediated endocytosis, a process attenuated by specific inhibitors. The RDT-NPs were then employed for targeted delivery of irinotecan, a chemotherapeutic agent, to cells overexpressing HB-EGF. Results indicate that the receptor-mediated uptake of RDT-NPs significantly enhances the efficacy of irinotecan in these cells [36].

#### Scavenger Receptors

Scavenger receptors are glycoprotein-based receptor-based superfamily is divided into various subclasses based on their structure and function. This receptor was earlier referred as a macrophagic receptor with a significant role in transcytosis-based lipoprotein uptake. These receptors possess higher binding efficiency with multi-anionic substrate and ligand-based assimilation. Scavenger cells such as macrophages, phagocytes, microglial cells and dendritic extension are the most common sites for the presence of these receptors [37]. Scavenger receptor class F member 2 (SCARF2), also known as scavenger receptor expressed by endothelial cells 2 (SREC-2), exhibits prominent expression in endothelial cells, characterized by extensive cytoplasmic domains. In contrast to its counterpart, SREC-1, which is well documented for its pivotal role in binding and endocytosis of diverse endogenous and exogenous ligands, SCARF2 has received limited attention, particularly in the context of modified low-density lipoprotein internalisation. Kim and coworkers [38] conducted a study that delves into the expression patterns of SCARF2, particularly in glioblastoma (GBM), revealing heightened expression levels compared to normal brain tissue. Utilizing The Cancer Genome Atlas database, comprehensive analysis underscores the widespread expression of SCARF2 in GBM, with elevated SCARF2 levels correlating with an unfavourable prognosis amongst glioma patients. These findings propose SCARF2 as a prospective diagnostic marker and therapeutic target in various cancers, including glioma. This research contributes valuable insights into the potential utility of SCARF2 in the realm of cancer diagnosis and treatment [38].

#### Efflux Receptor

The extent of drug transportation is mainly hindered due to the presence of P-gp 1 situated at the luminal site of endothelial cellular tight junction and astrocytic foot projections. P-gp 1 is an ATP-mediated efflux mediator involved in absorption, transmission, digestion and removal from circulation. Various in vitro and in vivo results have emphasized the importance of P-gp substrate inhibition restricting the transportation of various therapeutic molecules within central brain compartment. A description of P-gp 1 substrate and inhibitors currently available are enlisted in Table 1. Hence, conclusively various inhibitors and substrates simultaneously interact with the P-gp pump. This interaction evidently suggests the coadministration of P-gp substrate molecules and inhibitors for inhibition or stimulation of this transporter and thereby enables higher bioavailability within brain compartment. The rate of physiological barriers to allow passageway for therapeutic molecules is also assisted with ATP-binding cassette. Together these transporters transport drug molecules via endothelial bridges towards peripheral circulation. The BBB is impermeable for not only neurotherapeutic agents but also essential anticancer agents, and it also avoids uptake of other nutritional micromolecules. P-gp portal is selectively open for small-sized protein and biomolecules that can easily traverse through the BBB via concentration-dependent passive diffusion mechanism mainly based on lipophilic nature, molecular weight and surface charge. High molecular weight entities with adequate hydrophilicity like sugar and derivatives, amino acids, derivatised proteins and antibodies are unable to gain access via P-gp portal [39].

**Table 1:** List of P-glycoprotein substrates and inhibitors [40]

**P-gp substrates**
Cancer drugs (doxorubicin, daunorubicin, vinblastine, vincristine, paclitaxel, etoposide)
Lipid-lowering agent (lovastatin)
Cardiac drugs (digoxin, quinidine)
Antidiarrheal agent (loperamide)
Immunosuppressive drugs (cyclosporin A, FK506)
Steroids (aldosterone, cortisol, corticosterone, dexamethasone)
Antigout agent (colchicine)
Anti-tuberculosis agent (erythromycin, rifampin)
Anthelminthic agent (ivermectin)
Fluorescent dye (Rhodamine 123)
HIV protease inhibitor (amprenavir, indinavir, nelfinavir, ritonavir, saquinavir)
**P-gp inhibitors**
Cyclopropylibenzosuberane LY335979
Calcium channel blocker (verapamil)
Acridinecarboxamide derivative GG918 (GF120918)
Peptide chemosensitizers (reversin 121, reversin 125)
Progesterone antagonist (mifepristone (RU486))
Immunosuppressant (cyclosporin A, valspodar (PSC833))
Anti-arrhythmic agent (quinidine)
Antifungal agent (ketoconazole)
Topoisomerase (Xenova (XR5944))

The P-gp inhibitors hinder drug outflow and aid the substrate molecules to gain access within brain compartment. P-gp substrates enhances drug absorption, metabolism and excretion

## Molecular Targets within the BBB

Since the inception of neurotherapeutics, target-oriented nanodelivery is directed towards cellular organelles such as glial targeting, mitochondrial targeting, Golgi-directed and even site-specific delivery which are explored.

### Microglia-directed Delivery

Neuroinflammation is a prime culprit of various neurodegenerative challenges, and CNS disorders are augmented by macrophages and glial cells. These cellular units perform the Trojan horse–mediated cargo delivery, due to their phagocytosis and activation mechanism in diseased conditions. Many nanocarriers are developed to selectively target macrophage- or microglia-directed delivery so the therapeutic cargo can bring within the diseased area and thereby release content. The macrophage or microglial targeting is mainly facilitated by particle size, surface charge (neutral/cationic/anionic) and hydrophilicity: lipophilic quotient and structural arrangement greatly impact on targetability, specificity and pharmacokinetics of therapeutic cargo. Understanding the mechanistic internalisation of nanoparticles by microglia or macrophage can provide great insights towards designing effective platforms with improved targeting efficiency. In vitro studies have suggested that clathrin-mediated endocytosis serves as the major pathway for microglial uptake of quantum dots and polymeric nanoparticles, regardless of the activation status of microglia. Hutter et al. [41] have evaluated urchin-imitating structural alteration in gold nanoparticles, and such structural orientation enhances the available surface area for binding. The confocal microscopic evaluations suggested these nanoparticles radially gained access within microglial cells compared to rod or spherically shaped gold nanoparticles [41]. Additionally, few interventions have targeted the differential mechanistic uptake pathway for activated and inactive microglia for elucidating current knowledge gap. Particularly, for in vitro analysis, lipopolysaccharide-activated microglia revel higher cellular uptake efficiency of polyethylene glycol and poly(ε-caprolactone) [42]. Under an in vivo GBM model, tumour-associated microglia (TAM) are crucial in cellular internalisation of G4-PAMAM dendrimers within tumour microenvironment upon IV administration, and we observed profound deposition in TAMs with fluorophore-tagged nanocarriers distributed uniformly within the tumour growth [43]. The extent of nanocarrier internalisation via the activated microglia can be confirmed by the accumulation of nanocarriers within collateral spaces of tumour growth in tumour-bearing rodent models. These studies highlighted the apparent nanocarrier internalisation within the allied hemispheres of GBM rat model, whereas in healthy rodent animals, the net apparent particular internalisation remains zero due to non-activated microglia [44]. The microglial targeting mainly focused on the two pathways either via harmonisation via microglial activation with signalling pathways or controlling the healthy microglial population or avoidance of macrophage-based infiltration.

### Mitochondrial Targeting

Mitochondrial dysfunction is an epicentre of various neurological manifestations, providing a continuum for neuronal network and its interaction with mitochondrial oxidative stress. Mitochondria mainly catalyse electron transport chain and yield ATP molecules for the physiological functioning of cellular homeostasis. In neurogenerative diseases, the electron transport chain–mediated ATP synthesis undergoes massive shift and thereby damages the healthy physiological outlook of mitochondria. Mitochondrial drug targeting can be attained using following strategies: (i) by exploiting mitochondrial membrane potential, (ii) utilizing mitochondrial membrane-responsive lipid components and (iii) stealth nanotheranostic approach directed towards mitochondrial trafficking pathway. Mitochondria are an exceptional cellular organelle in terms of structural orientation and charge density. The membrane polarisation is derived from the inner mitochondrial membrane (IMM) potential, i.e. 150–180 mV. Highly lipophilic cationic molecules can easily traverse through hydrophobic lipidic bilayer membrane owing to cationic charge delocalisation. As per the Nernst equation, at room temperature, probability of passive translocation of cations accumulates ~ tenfold per 60.5 mV IMM potential distinction. The targeting efficiency increases based on the potential difference of applied magnitude. Therefore, positively charged nanocarriers can offer mitochondrial selectivity and specificity. For neuronal cells with resting membrane potential of nearly 60–80 mV, cation-conjugated nanocarrier accumulates several folds in cytosolic environment compared to extracellular compartment and similar correlation is reported in cytosol compartment versus mitochondria. The quantity of therapeutic moiety reached at IMM in vivo differs substantially, depending on cell orientation, physicochemical properties of conjugated therapeutic cargo, treatment interval and population of mitochondria. The translocation of mitochondria-directed cations depends on passive electrochemical gradient–driven pathway, and disease conditions often provide unprecedented physiological complications which further cause complexes in the mitochondrial targeting. Most well-recognized mitochondrial targeting prototype cations are triphenylphosphonium (TPP), and its chemical derivatives are explored for mitochondrial targeting. Most experiments employing TPP-conjugated nanocarriers focused on encapsulation of lipophilic molecules (quercetin, ubiquinone and tocopherol) and superoxide dismutase (SOD)-1 mimicking therapy. The partitioning coefficient of lipophilic TPP relies on mitochondrial membrane potential. Metal-induced electron transport chain (ETC) dyshomeostasis often ends up in proton gradient dissipation, profoundly reducing the matrix penetration efficiency of TPP-conjugated nanocarrier. MitoQ is another ubiquinone-based potent mitochondrial targeting agent showing reduced sensitivity towards cells deprived with proton gradient potential. Importantly, the net delocalisation of TPP and other cations across IMM into the matrix may consume charge gradient and may lead to complication of disruption of ATP synthesis and electron chain transport inhibition. Few organically derived antibiotics revel profound affinity towards cellular membranes, and similar molecular arrangement enhances the affinity coefficient of these molecules to IMM components such as mitochondria-attracted phospholipid, cardiolipin (CL) and lipid backbone–containing molecules. Unlike above-mentioned lipophilic cationic linkers that follow potential gradient and partitioning behaviour mainly into mitochondrial environment, CL-conjugated compounds are believed to be potential gradient-insensitive and reside within IMM. Therefore, IMM is a versatile mechanistic target which may superiorly address mitochondrial targeting. The Szeto-Schiller (SS) peptides, gramicidin S (GS) and hemi-GS are main examples of affinity-based localisation therapy. The hemi-GS peptide can connect via flexible anchor to molecule of choice without hampering the therapeutic payload, mechanistic activity and affinity with mitochondrial proteins. In contrast to GS, hemi-GS opted out for membrane permeabilization without antibacterial effect. Hemi-GS conjugates primarily concentrate at mitochondrial junction and IMM. Hemi-GS amino derivative, one of such derivatised conjugate, was reported for its safety, quick penetration across the BBB and residence in IMM in potential gradient-independent manner. Hemi-GS is a flexible peptide with adaptable chain length, and concentration of hemi-GS to drug-loaded conjugate can alter mitochondrial targetability efficiency, drug loading, surface charge, lipophilic quotient and hydrophilic region. Available preclinical studies suggest that hemi-GS derivatives are effective at attenuating oxidative stress and protection neurocognitive health. The SS peptides are affinity-mediated mitochondria-specific peptides. This peptide mainly aligns in the IMM and irrespective of its cationic origin, and a very small fraction of peptide localises based on proton gradient–driven mechanism. The SS possesses similar penetration ability, affinity towards IMM and proton gradient–driven mechanism These agents have been specifically utilized for inherent antioxidant effect, and various studies have supported their application as a drug delivery voyage system [45].

### Neuron Targeting

Neurons are the chief excitable electric-charged cells in the cerebral compartment that regulate and circulate signals to neurons via synaptic transmission. Despite their low apparent concentration, neurons are crucial targets for therapeutic cargo delivery as therapeutic targets in neurological or brain manifestations. The neuron-directed drug delivery is challenging as many factors need to be taken into consideration while designing such versatile drug delivery regimen. As neurons contribute to only up to 10% of brain volume, heterology in neuronal population and non-phagocytic nature of neurons are the common challenges imposed in targetability. While developing neuron-directed nanotherapeutics, the carriers should selectively target the diseased neuronal network without disturbing the homeostasis of other neurons [46]. Despite all the complexity, neuron offers various characteristic structural and molecular targets for developing nano-voyages. Under one such study, lipid nanoparticle–based neuronal internalisation is facilitated by ApoE-mediated astrocytic-dependent or LDL recognition. Particles undergo endocytosis and thereby attain neuronal uptake [47]. Neurons chemically possess higher concentration of sphingolipids, phospholipids, macromolecules and gangliosides. Tet1 is an amino acid–rich peptide that provides flexibility and binding affinity to targeting carrier system and exhibited efficient binding efficiency to neuronal cells. The neuronal phospholipid membrane is also enriched with neuronal cell adhesion molecule (CAM) that acts as an anchor for virus access via Pgp-1 binding acting as neuronal virus infiltrations. In an another study [48], small interfering RNA (siRNA)-based nanocarriers were modified with CGN and Tet1 peptide for enhanced brain and neuronal uptake, respectively. The prepared nanocarriers exhibited caveolae and clathrin-mediated endocytosis, successfully translocated from the lysosomal hemisphere and gained access within neuronal cytoplasm. Additionally, it successfully reduced agglomeration of senile plaques and also provided neuroprotection and neurogenesis [48].

## Formulation-based Blood–Brain Barrier Traversing Methodologies

Various formulations have been already developed for a variety of neurological sequelae and traversed across the BBB for successful CNS targeting. Herein, various approaches for BBB navigation are discussed.

### Intranasal Therapeutic Delivery

Intranasal administration is an appealing approach as it offers noninvasive method and easy approach for successful targeting and transportation of drug via the BBB hurdle. Intranasal drug delivery mainly travels through nasal mucosa, olfactory basal nerve and other connective tissues along with neurons and axonal extensions. The prime advantages associated with the intranasal delivery are avoidance of first-pass metabolism, peripheral degradation kinetics and off-targeted drug lodging. Till date, various preclinical and clinical data are reported for intranasal drug administration and BBB targeting [49]. The total available mucosal surface area within olfactory area is limited in humans whereas higher surface availability can be seen in rodent and animal models. Hydrogels or mucoadhesive excipient-based formulation via intranasal route is already tested under various experiments. Surface modification and conjugation with peptide, antibodies and ligands have improved the targeting ability and less irritability via intranasal administration. In recent years, drug delivery via intranasal pathway is widely utilized and developed as a versatile approach for drug delivery [29]. Wang et al. [50] have reported temozolomide (TMZ)-conjugated gold nanoparticles that are functionalized with an antibody against ephrin type-A receptor 3 (anti-EphA3-TMZ@GNPs) for intranasal delivery. The synthesized anti-EphA3-TMZ@GNPs demonstrated commendable safety in a nasal mucosa toxicity assay. In vitro investigations unveiled a conspicuous escalation in cellular uptake and toxicity of anti-EphA3-TMZ@GNPs in relation to TMZ, manifesting a superior cell apoptosis ratio against C6 glioma cells. Furthermore, assays conducted on TMZ-resistant glioma cells (T98G) divulged that the IC50 of anti-EphA3-TMZ@GNPs was markedly lower by a factor of 18.5 compared to TMZ. Western blot analyses indicated a proficient downregulation of *O*-6-methylguanine-DNA methyltransferase expression by anti-EphA3-TMZ@GNPs, consequently enhancing the chemosensitivity of T98G to TMZ. The in vivo efficacy was evaluated in rats bearing orthotopic gliomas, revealing that anti-EphA3-TMZ@GNPs protracted the median survival time to 42 days and substantially heightened tumour cell apoptosis in contrast to TMZ. In summary, anti-EphA3-TMZ@GNPs represent a promising intranasal drug delivery modality for the effective treatment of GBM [50].

In another study, nanoparticles co-modified with borneol and lactoferrin (Lf-BNPs) encapsulating dopamine were investigated for intranasal delivery to optimize therapeutic efficacy and minimize side effects in Parkinson’s disease (PD). Dopamine Lf-BNPs fabricated utilizing the double emulsion solvent evaporation revealed that the application of dopamine Lf-BNPs exhibited relatively low cytotoxic effects in SH-SY5Y and 16HBE cells. Qualitative and quantitative examinations of cellular uptake indicated that lactoferrin (Lf) modification of nanoparticles augmented the cellular uptake in SH-SY5Y and 16HBE cells, while borneol modification enhanced the cellular uptake specifically in 16HBE cells. In vivo pharmacokinetic investigations demonstrated a significantly higher (*p* < 0.05) area under the concentration–time curve (AUC_0_–12 h) in the rat brain for dopamine Lf-BNPs compared to dopamine nanoparticles. Intranasal administration of dopamine Lf-BNPs effectively ameliorated 6-hydroxydopamine-induced striatum lesions in rats, as evidenced by the contralateral rotation behaviour test and assessments of striatal monoamine neurotransmitter content. Collectively, intranasal administration of dopamine Lf-BNPs emerges as a promising drug delivery system for PD [51]. One significant challenge associated with nasal mucociliary clearance has been effectively mitigated through the formulation of advanced mucoadhesive nanocarriers. However, several additional challenges impede the progression of this platform towards becoming a viable end-user product.

### Invasive Brain Administration

Intracerebral infusion administration is an invasive BBB targeting strategy involving direct administration of drug within brain endothelial cells. Direct instillation of drug within neural parenchyma offers advantages like site-specific dosage loading, smaller dose and improved circulatory half-life. Das et al. [52] investigated intracerebral injection of γ-linolenic acid (GLA) on normal dog brain and in 15 patients with malignant gliomas. Histopathological examination showed that GLA did not cause cytotoxicity to the normal dog brain cells. Administration of 10 mg of GLA to glioma patients via a cerebral reservoir placed in the tumour bed, at the rate of 1 mg/day over a period of 10 days, revealed that GLA was not only safe and nontoxic but could also regress cerebral gliomas as evaluated by computerised tomography and increased survival of the patients by 1.5–2 years [52]. Despite all the advantages offered by intracerebral infusion, its application is still restricted due to chances of parenchymal damage, heightening of intracerebral pressure, cerebral fluid discharge from site of injection, intracerebral infections, brain haemorrhage and low patient acceptability. Owing to these factors, the applicability of this method is limited.

Effective therapeutic intervention in AD is impeded by the formidable BBB, posing challenges to drug penetration, and the nonselective dispersion of therapeutic agents in the brain. Furthermore, the intricate pathophysiological mechanisms of AD encompass various pathway dysregulations, limiting the efficacy of singular therapeutic agents. In this context, a nanoparticle system, incorporating dendrigraft poly-l-lysine (DGL)-based siRNA and D peptide (Dp), has been engineered to address these challenges. The designed nanoparticle aims to selectively target and traverse the BBB, penetrate the brain parenchyma and accumulate specifically at AD lesions. Within this system, T7 peptide, known for its specific affinity to transferrin receptors on the BBB, is tethered to DGL via acid-cleavable long polyethylene glycol (PEG), facilitating enhanced internalisation, prompt escape from endo/lysosomes and efficient transcytosis. Subsequently, Tet1, with specific targeting capabilities towards diseased neurons, is conjugated onto DGL using a short PEG linker. Upon exposure, Tet1 facilitates nanoparticle transport to AD lesions, facilitating drug release. Consequently, the formation of Aβ plaques is suppressed. Moreover, the neurotoxic effects induced by Aβ plaques and tau protein phosphorylation (p-tau) tangles are alleviated, leading to a substantial improvement in cognitive function in AD mice. In summary, this system exhibits a programmable capacity to target both the BBB and neurons, thereby significantly enhancing intracerebral drug accumulation and augmenting the efficacy of AD treatment [53].

### Interim Blood–Brain Barrier Disruption Methods

A short-term BBB appendageal disruption is being tested under various preclinical trials for successful drug delivery methodologies to surpass the BBB hurdle.

#### Osmotic BBB Disruption

Osmotic agents like fructose, mannose, glucose, amides, glycerol and urea induce osmotic imbalance that causes partial BBB opening for short interval. For example, mannitol is being used as a BBB disruptor via endothelial dehydration–based shrinking of tight junctions. However, higher concentration of osmotic agents may lead to irreversible cerebral damage, as higher osmosis leads to hyperpermeability which can damage myelin and neuronal network. The major challenge associated with this technique is that it may allow entry of microbes and pathogens and can also lead to sudden epileptic shock or oedema. Various surfactants and solvents are also reported for BBB interruption; for instance, ethanol, poloxamer 188, glycerol and dimethyl sulfoxide are used for BBB relaxation and facilitation of drug uptake [54]. In the context of BBB disruption in response to lesion stimuli, intracellular tension emerges as a pivotal factor influencing the integrity of tight junctions, particularly affecting structures like occludin and ZO1. Li et al. [55] employed a fluorescence resonance energy transfer (FRET)-based tension probe and cpstFRET analysis to assess intracellular tension for occludin and ZO1. Changes in the mobility ratios of occludin were evaluated through the fluorescence recovery after photobleaching (FRAP) test. Cytoplasmic osmotic pressure (OP) was quantified using an osmometer, and the count rate of cytoplasmic nanoparticles was measured using NanoSight NS300. In vivo BBB permeability was determined by monitoring changes in Evans blue (EB) injected into Sprague Dawley rats. Tight junction formation was assessed through the measurement of transendothelial electrical resistance (TEER). Intracellular calcium or chloride ions were measured using Fluo-4 AM or MQAE dyes. BBB lesions correlated with alterations in occludin/ZO1 tension. Increased intracellular osmotic pressure was implicated in modifying BBB permeability, potentially through microfilament or microtubule depolymerization and the substantial production of protein nanoparticles, in accordance with the Donnan effect. The restoration of osmotic pressure related to protein nanoparticles effectively reversed the effects of changes in occludin/ZO1 tension in the context of BBB lesions. Outward tension of intracellular osmotic potential also induced an upregulation of membrane fluidity, facilitating nonselective drug influx. These findings delineate a crucial mechanical mechanism underlying BBB lesions, with protein nanoparticle–related osmotic pressure emerging as a prospective therapeutic target for conditions associated with BBB lesion–induced brain diseases [55].

#### Stimulus-based Blood–Brain Barrier Disruption

##### Ultrasound-Driven Blood–Brain Barrier Disruption

Ultrasound waves can be used for partial disruption of BBB tight junctions. Such disruption can be achieved by microbubble-induced ultrasound oscillation at a focused time interval. This technique beholds great opportunity in BBB permeability. There are various studies on prefilled gas bubbles for partial rise in permeability which facilitates the brain-targeted delivery. This allows focusing on a contained spot in brain with the help of acoustic energy and allows dissemination within neural compartment. The ultrasound waves supplied on the transducer surface, while the piezoelectric element shifts transduction energy in mechanical energy. Earlier ultrasound disruption involved thermal method or gas bubble formation which leads to development of cavitation at the transducer site. A persistent demand exists for noninvasive methodologies enabling precise manipulation of brain activity concerning molecular, spatial and temporal parameters. Rich et al. [56] have investigated the utility of magnetic resonance imaging (MRI) visible nanoclusters based on albumin for targeted and time-specific drug delivery to the rat brain. The evaluation of deposition of intravenously injected nanoclusters into specific brain regions through focused ultrasound-mediated blood–brain barrier opening was conducted. In vivo confirmation of nanocluster localisation was achieved using MRI. Subsequently, upon confirmation of nanocluster delivery, a second focused ultrasound treatment, conducted in the absence of circulating microbubbles, triggered the release of the nanocluster payload into brain tissue. Notably, glutamate release from nanoclusters in vivo led to heightened c-Fos expression, indicative of the nanoclusters’ sufficient loading capacity to induce neuronal activation. This innovative technique for noninvasive stereotactic drug delivery to the brain with temporal precision holds promise as a novel approach for preclinical in vivo investigation of brain circuits, with considerable implications for clinical translation [56]. Song et al. [57] have shown augmented efficacy of TMZ against GBM using liposomal TMZ formulation (TMZ-lipo) locally administered into GBM through the utilization of ultrasound-mediated BBB opening technology. This approach effectively curtailed tumour growth and prolonged the survival of animals bearing GBM, with no significant discernible side effects compared to control rats [57].

##### Magnetically Driven Blood–Brain Barrier Disruption

Ficiarà et al. [58] explored magnetic oxygen–loaded nanobubbles (MOLNBs) as theranostic carriers designed for the delivery of oxygen and chemotherapy to brain tumours. The MOLNBs are synthesized by incorporating superparamagnetic iron oxide nanoparticles (SPIONs) onto the surface of polymeric nanobubbles. The investigation encompasses an examination of physicochemical attributes, cytotoxicological properties, in vitro internalisation by human brain microvascular endothelial cells and the mobility of MOLNBs in a static magnetic field. MOLNBs exhibit a secure profile as oxygen-loaded vectors capable of traversing brain membranes and navigating the CNS to transport payloads to specific regions of interest. Additionally, MOLNBs are traceable through either MRI or diagnostic ultrasound (US). The potential applications of MOLNBs lie in the targeted delivery to brain tumours, where they can enhance conventional radiotherapy and facilitate chemotherapy release under the influence of customized magnetic fields, all while being monitored via MRI [58].

### Implants

Cerebral synthetic implants are recent trends in the treatment of neurodegenerating ailments. These implants are installed in the CNS via surgical procedure for the controlled and systematic release of medicaments. These implants can alter the homeostasis around the area of implantation. Bennett et al. [59] have developed the hydrogel containing polymer-based implant for treatment of ischaemia-induced stroke with BBB refurbishment and avoidance of untoward off-target drug delivery. Brain-derived neurotropic factor was targeted for extended release via hydrogel matrix. Rats were administered with vehicle, neurotropic factor–administered group was administered with low neurotropic factor containing hydrogel-encapsulated implant and high neurotropic factor containing hydrogel-encapsulated implant and all the groups were primarily instilled implants at the site of infarction 8 days after the completion of dosing. Incidence of infarction was reported lowered in rats administered with higher neurotropic factor containing hydrogel, with levels of phagocytosis as well as astrocytic and microglial activation higher than those in other groups [59]. Reported implants for brain-targeted delivery are listed in Table 2.

**Table 2:** Implantable drug delivery system for brain targeting [60]

Disease condition	Active ingredient	Description of system	Outcome of experiment
Brain tumour	Methotrexate, cetuximab	Cetuximab conjugated with the polyamidoamine dendrimer tail loaded with the methotrexate	This biconjugate implant showed higher biodistribution and affinity with the EGFR
Brain cancer	Carmustine, cyclophosphamide, paclitaxel	Carmustine, cyclophosphamide/paclitaxel loaded in polyanhydride polymer	Pharmacokinetic profiling reveals that carmustine achieves 1000 times higher concentration within a brain compartment
Stroke	Growth factor	Polyethylene glycol modified methylcellulose and hyaluronan	Epicortical deposition of hydrogel revelled highest progenitor cell proliferation along with its stimulation
Alzheimer’s disease	Nerve factor	Nerve factor loaded in self-degradable porous silicon oxide	Prepared silicon chips implanted in rats for 8 weeks, microparticles penetrate within brain capillaries and higher localisation shown within brain compartment
Parkinson’s disease	Tannic acid	Tannic acid–coated nanogold cross-linker as an injectable implant	The prepared hydrogel revelled improved self-healing and antioxidant ability. Histological evaluation showed higher tyrosine hydroxylase–enriched neurons and lowered inflammation

Implantable devices are widely used in the treatment of neurological sequelae. The amalgamation of nanotechnology and implants have showed various applications in BBB targeting

### Prodrug Delivery

Prodrugs are a partially active or inactive agent synthesized via chemical stabilization of medicaments. Prodrugs possess higher lipophilicity, this characteristic improves the brain permeability and, once it gains access within neural environment, it converts to active drug via enzymatic degradation. Prodrug approach allows easy brain transcytosis via efflux transportation mechanism. More often, prodrugs coupled with efflux inhibitors are an approved strategy for prodrug delivery. Mostly prodrugs are accompanied with elacridar, laniquidar and tariquidar as a P-gp inhibitor for enhancement in prodrug delivery approach. Such prodrug approach is explored mainly in case of l-dopa delivery within brain along with codrug approach via O-methyltransferase catechol inhibitor. l-Dopa is often coadministered with entacapone for potent brain delivery with longer circulation interval. Lalatsa and coworkers [61] developed a neuropeptide strategy for brain targeting of leucine-enkephalin peptide and palmitic-based prodrug encapsulated with chitosan delivery via oral administration. Pharmacokinetic evaluations revealed improved brain uptake by more than 60% with prolonged antinociceptive activity. This preclinical analysis improved prodrug delivery via peptide stabilization in plasma across the BBB [61]. In another report by Ju and colleagues [62], hyaluronidase-targeted hyaluronic-based and doxorubicin-based nanoparticles were constructed for brain targeting. Such co-modified nanoparticles showed better targeting ability at the BBB and enhanced overall survival rate within mice for cranial metastatic breast cancer. Such prodrug approach can improve the scenario in brain carcinoma [62].

#### Peptides

Peptides which originated from smaller amino acid segments interconnected via peptide linkages have received significant attention as a neuroprotective entity. Peptides have displayed contrasting therapeutic pathways; the action of peptides initiates with cellular adhesion or relevant protein molecules. Peptides usually possess higher specificity and predefined metabolism pathway with shorter circulation period. Peptide application as therapeutic entity is limited due to physical, chemical and enzymatic metabolism accompanied with poor intestinal membrane permeability. Such factors have narrowed the overall bioavailability of peptides via oral administration. Parenteral route of administration is a usual method applied for delivery of neurotherapeutics, due to its targeting predictability and improved bioavailability. Nonetheless, a repeated administration due to shorter half-life has reduced the patient acceptability. In terms of oral drug delivery, peptide must pass through intestinal membrane first and later needs to gain access through the BBB portal to reside within neuronal compartment. Other convenient routes such as sublingual, intranasal, buccal, ocular, rectal and intraperitoneal have been tested for peptide delivery [63]. Peptides utilized in various preclinical experiments are enlisted in Table 3.

**Table 3:** Methods for peptide delivery across the BBB [64]

Peptide	Formulation	Route of administration	Disease
Neurotensin	Angiopep-2 ligand	IV	Allodynia
Dalargin	Polymeric nanoparticles	IV	Antinociception
Opioid peptide	VH0445 peptide conjugate	IV	Antinociception
Aurimmune	Tumour necrosis factor conjugate	IV	Solid tumour
Leucine-enkephalin	*N*-Palmitoyl-monomethyl-*N*,*N*-dimethyl-*N*,*N*,*N*-trimethyl-6-*O* glycolchitosan ligand	Oral	Solid tumour
*O*-Palmitoyl tyrosinate ester drug (dalarg)	Nanofibers	IV	Antinociception

Above-mentioned peptide-based delivery is reported in BBB targeting via different routes of administration

#### Antibodies

Monoclonal antibodies are employed for successful delivery of drug molecules, protein supplements and peptide-based active agents. Hybridoma technique allows isolation of monoclonal antibodies with desired specificity, and such systematic development leads to production of immune-specific antibodies. Monoclonal antibodies possess longer circulating half-life, higher molecular weight and high water solubility. Such characteristics compromise the assimilation of antibody via simple diffusion transcytosis, although receptor-directed transcytosis is the most widely used approach for brain-targeted delivery. These receptors are also present at other sites or organs with a few or limited site availability at CNS junction. Transferrin is highly aggravated in brain region under endothelial junction, making transferrin a versatile targeting portal compared to other receptors. In recent years, OX-26 is being used widely for transferrin-specific monoclonal antibody delivery across the BBB junction. This antibody targets mainly glycoprotein fraction of transferrin receptor without competing with transferrin substrate. Various immunohistochemical and in vivo imaging data have confirmed the passageway for antibody for BBB targeting [65]. Aktaş and coworkers [66] synthesized chitosan nanocarrier capped with OX-26 antibody for treatment of cerebral ischaemia. OX-26 and chitosan possess higher affinity with transferrin receptor further tagged with fluorescent agent. The outcome reveals significant amount of nanocarrier reached within neural compartments owing to OX-26 peptide–based transportation within brain [66]. Kuo and Ko [67] developed saquinavir-loaded SLNs grafted with 83–14 monoclonal antibody. Prepared nanoformulation was analysed in HBMEC cell line for understanding the endocytosis mechanism by staining nuclei and insulin receptors. Further phagocytosis mechanism was analysed via RAW246.7 macrophages, and 83–14 monoclonal antibody concentration improves permeation across the BBB and reduction in phagocytosis extent within macrophages [67]. Various antibodies reported for brain-targeted delivery are enlisted in Table 4.

**Table 4:** Role of antibodies in brain drug delivery [68]

S.No	Antibody	Biomarker	Disease	Key points
1	Bispecific antibodies	Transferrin receptor and Aβ peptide	Alzheimer’s disease	BI 1034020 specifically targets the Aβ (A40 and A42) epitopes
2	BIIB033, anti-LINGO-1 mAb	LINGO-1	Multiple sclerosis	Strong affinity and specificity for LINGO-1, causing remyelination in MS
3	HMGB1 targeting mAbs	HMBG1	Neuroinflammatory diseases	Neuroinflammation treatment with A25–35 was lessened by HMGB1, and this included the activation of the receptor for an advanced glycosylation end product (RAGE)
4	Bapineuzumab and solanezumab	Soluble form of Aβ plaque	Alzheimer’s disease	Cognitive deterioration in moderate AD patients is alleviated
5	Camelid–VHHs	Amyloid peptide and tau neurofibrillary tangles	Alzheimer’s disease	Distinguish especially intracellular tau neurofibrillary tangles and extracellular amyloid plaques
6	Ofatumumab	CD20 on lymphocytes	Multiple sclerosis	Therapy for MS relapses
7	Aducanumab and gantenerumab	Insoluble and aggregated Aβ plaque	Alzheimer’s disease	Attaches to protofibrils
8	Natalizumab	Alpha-4 integrin	Multiple sclerosis	Inhibits the interaction of VCAM-1 with 41-integrin by binding to it
9	LCN2 targeting Abs	LCN2	Cerebral ischaemia, stroke and brain injury	Minimize the brain damage caused by LCN2

Recently, various antibodies are utilized for targeting specific biomarkers for neurodegenerative diseases

## Interplay of Blood–Brain Barrier and Neurological Sequelae

**Fig. 5:**
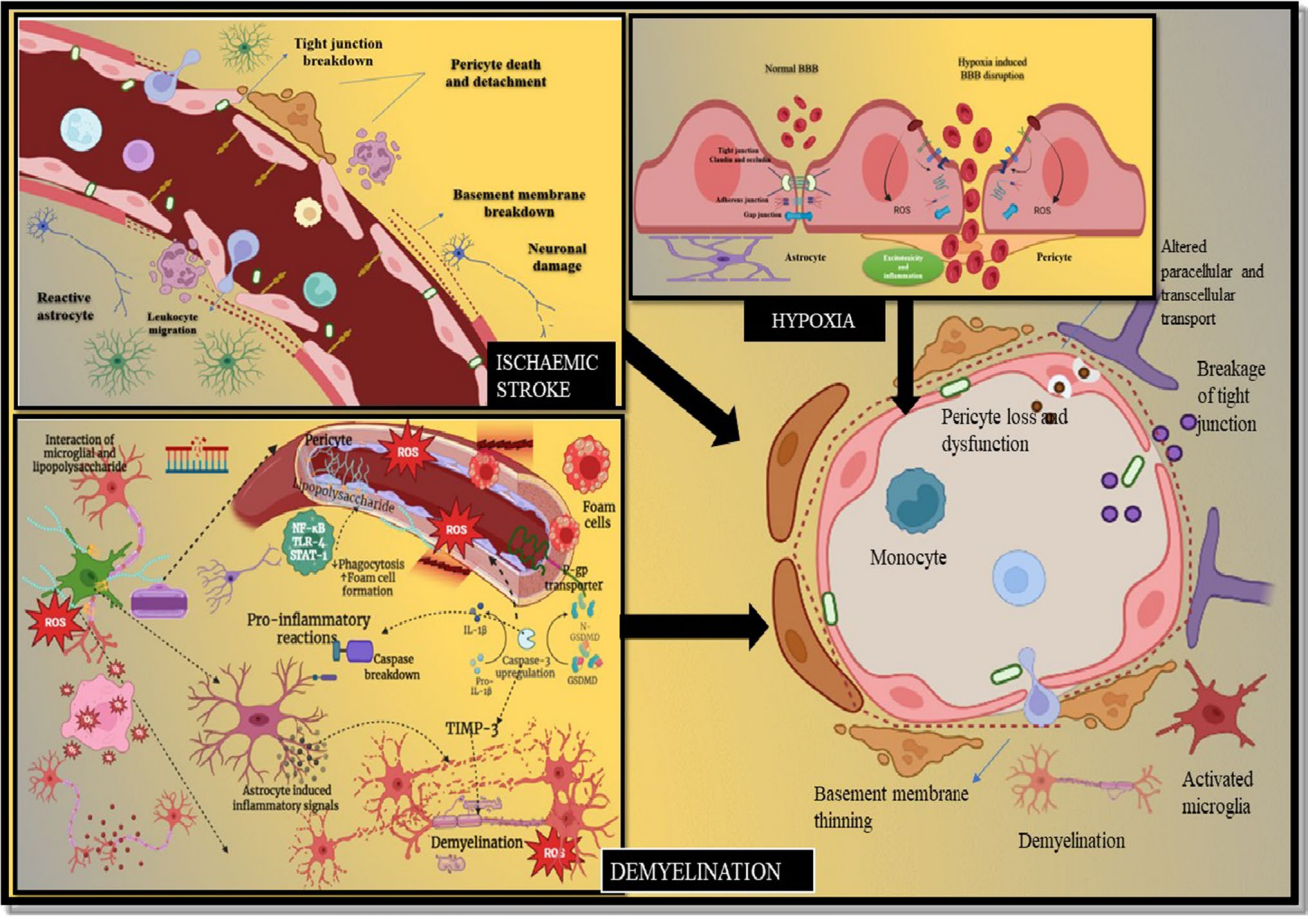
Depiction of neurological sequelae and its impact on BBB integrity. Ischaemic stroke, demyelination and hypoxia are the common neurological challenges that alter the permeability of the BBB or often disrupt its integrity. All these neurological challenges are resultant of involvement of proinflammatory mediators and hyperactivation of immune system. BBB damage is usually accompanied with damage in tight intracellular cells, hyperactivation of glial cells and altered permeability quotient via paracellular or transcellular pathway

Role of neurological challenges and its impact on integrity of the BBB are represented in Fig. 5.

### Myelin Mutilation

Myelin is the outer covering of nerve fibre endings associated with neuron sheathed with white matter termed as white matter or broadly termed as white matter. Myelin damage is associated with brain damage in developmental stage which further escalates disturbances in oligodendrocytic development, formation of cystic lesions within myelin and demyelination within different regions of brain. Although myelin mutilation is observed in younger children, but in later stages, further injuries or infections may aggravate deprivation in grey matter. Myelin deprivation is the leading cause for development of neurological impediment in infants and toddlers and the prime cause for cerebral palsy, motor neuronal diseases and autism. In various lines of experimental models, prime causative factors for chronic myelin damage are often associated with overexpression of cytokines, free superoxide molecules, high administration of heavy metals and enhanced neurotransmitter synthesis or release from synaptic cleft. Clinical data supports ischaemia-induced pathological development of demyelination at younger age [69].

### Hypoxia-induced Encephalopathy

Hypoxia-mediated encephalitic infections are reportedly linked with the leading cause of brain damage in toddlers and neonates. Currently, there are no available biomarkers for early diagnosis of encephalitic infection in neonates. Owing to such limitations, neonates are currently treated with hypothermic methodologies for treatment of encephalitic reactions and improved life expectancies, but sudden death and severe reactions are inevitable in such therapeutic treatment. To avoid development of such challenges, preliminary precautions need to be taken under consideration for avoiding conditions that may involve deprivation of oxygen and essential nutrients. The neonate’s brain response towards hypoxia and ischaemia is a sequential procedure primarily initiated with higher cerebral blood flow and lowering in local vasoconstriction and thereby reduced cardiovascular output. Ischaemia is accompanied with higher neurotransmitter synthesis, mitochondrial permeation and production of superoxide radicals, and these factors allow progression towards apoptosis and brain oedema. Such multifactorial impact on BBB disruption led to dissemblance within tight junctions, occludin, claudin and other crucial epithelial cells within the BBB. Such events enhance the permeability or sensitization of brain towards inflammatory mediators, cytokine aggregation (tumour necrotic factors, interleukins, monocytic proteins, chemoattractant molecules, vascular endothelial factors, cell adhesion molecules) and astrocytes [70].

### Ischaemic Stroke

Stroke is a resultant of interruption of brain circulatory kinetics due to local damage or other substantial factors within vascular system. Perinatal stroke and postnatal ischaemic incidences are the leading causative factors for the cerebral BBB deterioration. Ischaemic stroke is a preliminary factor for worsening of cerebral palsy, epileptic shock, language dysphonia and autistic behaviour. Ischaemic stroke can lead to excess trafficking, leakage in barrier and infections via disrupted BBB [71].

### Traumatic Cerebral Damage

Traumatic cerebral–based damage in neonates or toddlers can give rise to challenging cognitive impairment compared to damage in adult brain, as probable reason is associated to maturation of cortex and protecting sutures as age progresses. Intracerebral haemorrhage of the germinal epithelium matrix floor led to permanent and irreversible damage within the brain. Children encountered with brain haemorrhage episodes are on the verge of ependymal damage and matrix-associated ventricular damage. Children going through such injuries are often at a higher risk of motor neuron diseases or permanent handicap state [72].

## Predisposing Pathological Factors for BBB Disruption

Maintenance of BBB integrity is a prime function for the protection and homeostasis of brain. Various molecules, proteins, peptides and antibodies are crucial for maintenance of BBB integrity.

### Morphological Mutation in Endothelial Cells

Matrix metalloproteinase proteins are crucial protein molecules assisting in embryonic cerebral development, organ development, blastocyst and bone calcification, and internal organ healing. Excessive metalloproteinases are often associated with damage to the tight endothelial junction containing occludin, claudin, cadherin and desmosomes cells and thereby degrade the BBB vasculature. This event facilitates the BBB infiltration, brain haemorrhage and cerebral oedema [73].

### Radical-Based Excitotoxicity

Stress-induced neurotoxicity in in vivo models was analysed for understanding the impact of stress mechanism on generation of free superoxide and thereby induction of neurodegeneration. Nitric oxide generation when subjected to stress factors escalates the BBB permeability and further loss in BBB integrity. Brain is composed of maximum lipid fraction that makes it more susceptible to free radical–based damage, thereby altering the permeability of the BBB. Increased encounter with free radicals induces excess oxygen uptake and formation of reactive oxygen species. In the presence of high concentration of free radicals, cerebral injury imposed on endothelial cells leads to modification in BBB integrity. Excessive synthesis of glutamate acts as a preliminary factor for neuron degeneration. Higher glutamate concentration can cause hyperactivation of ionotropic and metabotropic initiated signal cascade pathway for necrotic reaction and pre-apoptotic factors for further worsening of BBB integrity [74].

### Angiogenesis Reactions

Angiogenesis acts as a protective mechanism as it acts as an ischaemia-relieving mechanism in hypoxic regions. Chiefly TNF-α, IL-8 and growth factors are involved in the blood circulatory pathways essential for vasodilation and vascular growth. These factors are crucial for proliferation, migration, cellular adhesion and degradation of cellular matrix. The intravenous administration of VEGF has proven beneficial in various experiments as this resulted in lowering of cerebral injury, oedema and apoptotic cell quotient.

### Cerebral Oedema

Oedema is the most prominent cause of brain injury reported in neonates and toddlers. One of the probable reasons is higher accumulation of water within cerebral cortices, due to water dyshomeostasis within brain ventricles. Mutations in aquaporin channels can be the leading cause followed by CNS damage resulting in cerebral oedema in young children [75].

### Inflammatory Mediators

Neuronal inflammation is a primary aggravating cause for cerebral insults. Cerebral inflammation is the resultant of pathogenic or microbial invasion and thereby releases proteins from infected cells. Inflammation or invasion of such pathogen elicits activation of astrocytes, dendritic cells, endothelial cells and microglial appendages. Such activation of protein leads to production of proinflammatory mediators with increased overexpression of adhesion protein molecules linking TNF-α, IL-1, IL-6 and cytokines. Such inflammatory mediators catalyse BBB permeation extent, destabilization of tight junction and alteration in extracellular proteins and overall stability of endothelial basal membrane. Microglial cells are the prime cells that encounter with the pathological cells, and further pathogens transmit with astrocytic cells to the site of infection and release inflammatory cytokines. Synthesis of such mediators improves the sensitization of the BBB and allows access to various macrophages and other inflammatory cells from the systemic blood circulation. Various preclinical studies have been carried out to analyse the impact of neuroinflammation and brain injury in BBB disruption [76].

## Recent Nanotechnological Platforms for Blood–Brain Barrier-Targeted Drug Delivery

The development of nanotechnology through integrated multidisciplinary efforts leads to innovative understanding in neuronal compartment, methods for detection and therapy of brain illnesses. Considering the limits found in the drug vehicles towards the BBB, novel delivery platforms carrying therapeutic compounds are developed using nanomaterials, and these nanovehicles offer advantages like smaller size, biocompatibility, longer blood circulation and lack of toxicity. The targeted brain areas used by the nanotechnology-based medication delivery systems are dependent on both specific and general mechanisms. The progress associated with drug delivery via nanovehicles, such as NPs, dendrimers, liposomes, carbon nanotubes and micelles, received huge attention recently. These vehicles can deliver pharmaceutical drug molecule, peptides, proteins, nucleic acids or vaccines [77]. Small molecules with a high lipophilicity can passively diffuse across the BBB. The permeability and solubility of drug molecules are frequently closely associated with lipophilicity [78]. High lipophilicity can induce early metabolism, poor solubility and poor absorption. NPs must possess particular characteristics permitting the diffusion of a specific subset of chemicals. Drug delivery via the BBB is possible by the ability of nanocarrier to gain access through the tight connections separating the endothelial cells of the BBB. Moreover, the transcytosis and endocytosis of NPs can facilitate the transport of medication via the endothelial layer of cell. Lipid NPs can pass via the BBB by a variety of transfer mechanisms, such as receptor-mediated endocytosis, transcytosis and paracellular route based on the lipophilic quotient of cargo voyage. Various nanoformulations enhancing drug penetration towards the BBB are discussed as follows [79].

### Liposomes

The BBB can impede drug absorption in brain; however, liposomal composition is anticipated to circumvent these limitations. Phospholipids and cholesterol are combined to form small, spherical vesicles called liposomes, each of which contains one or more phospholipid bilayers. Liposomal components are biologically inert, biodegradable, non-immunogenic and with minimal inherent toxicity. Liposomes targeting transferrin receptors have been widely investigated for penetration of the BBB. Jhaveri et al. [80] developed anionic liposomes for delivery of resveratrol for GBM therapy. It was observed that surface modification of liposomes enhanced cellular uptake and inhibited cell proliferation in GBM cells. The researchers evaluated the effect of ligand density on a nanoparticle’s capacity to target and found an increase in cellular uptake with increased ligand intensity of 41.5 mol%. After the saturation of TfRs, the ligand intensity produced reverse effect. The studies performed in in vivo mice with subcutaneous tumour xenografts showed that tumour growth inhibition as well as the mice survival were enhanced on surface modification of liposomes with Tf [80]. Porru et al. [81] produced cationic liposomes and compared its cytotoxic potential with unmodified liposomes on human GBM cells. It was observed that both Tf-modified and unmodified liposomes increased the antiproliferative activity of drug. The pharmacological effectiveness of unmodified and modified Tf was evaluated using tumour xenograft models. In vivo imaging was utilized to detect the real-time biodistribution of liposomes. The acquired data showed that surface modification enhanced NP localisation inside tumour in both intramuscular and cerebral xenografts. Additionally, animals with both cerebral and intramuscular xenografts survived longer with Tf liposomes compared to unmodified liposomes, decreasing tumour growth and improving survival rates [81]. The anticancer drug cisplatin was delivered using cationic liposomes created by Lv et al. [82]. Cisplatin was encapsulated in Tf-modified liposomes and enhanced the inhibitory action of drug, according to cytotoxicity tests on GBM cells of rats. The scientists demonstrated that surface alterations significantly enhanced the transfer of liposomes over the BBB, with the use of a BBB model in vitro. Researchers also confirmed that Tf liposomes retained integrity following BBB permeability. The ability of liposomes to successively traverse the BBB and enter the GBM cells was further studied using an in vitro coculture model. Outcomes demonstrated that, in comparison to unmodified liposomes, Tf modification improved the delivery over the BBB and the ability to target C6 cells, resulting in higher antiproliferative properties [82]. For theragnostic applications, Sonali et al. [83] created Tf-tailored liposomes co-encapsulating quantum dots and the chemotherapeutic medication docetaxel. Surface modification brings extensive drug assemblage brain tissue after IV injection, according to biodistribution experiments conducted on rats, demonstrating the system’s capacity to target the brain [83].

Glutathione (GSH), a powerful antioxidant, is an intracellular tripeptide. GSH has recently been investigated for its transporter-dependent transcytosis-based uptake of liposomal nanosystems via MRPs including MRP1, MRP2 and MRP5 activation. Brain drug delivery using glutathione stealth liposomes has been shown to be safe and effective [84]. The improvement in carboxyfluorescein’s absorption in brain as a model drug was investigated by Rip et al. [85]. GSH liposomes boosted fluorescent trace levels in brain by many times more than plain PEGylated liposomes [85]. EGCG- and resveratrol (RSV)-encapsulated liposomes were proposed by Kuo et al. [27] therapy for PD treatment. According to immunofluorescence assays, in an in vitro BBB model using HBMECs and SH-SY5Y cells, leptin RSV-EGCG-based liposomes showed better permeation than non-modified liposomes [27]. By reducing apoptosis, expression of tyrosine hydroxylase and dopamine transporter is increased, and the nanocarrier further improved the ability of RSV and EGCG to heal damaged dopaminergic neurons. The cell-penetrating peptide TAT that penetrates cells quickly effectively crosses the BBB. To circumvent inadequate delivery of traditional formulations in the brain, unique TAT-modified liposomes (TAT-LIPs) were designed, and modified phospholipids serve as an anchor in formulations. In this study, the liposomes were created by covalently conjugating TAT with cholesterol. Rat brain capillary endothelial cellular uptake was investigated, and the TAT-LIP pathway during endocytosis was determined. In order to assess trans-endothelial potential to traverse the BBB and its transport mechanism, an in vitro BBB model was employed. The outcomes demonstrated that TAT-positive LIP’s charge significantly improved its brain delivery. One of the potential BBB-crossing pathways for TAT-LIP is absorptive endocytosis. Hence, TAT-LIP was a possible medication delivery method for the brain because of its high BBB transport efficiency [86]. By altering the liposome surface with the peptides DCDX and c(RGDyK), researchers created a liposomal formulation that could bypass various barriers. c(RGDyK) is an integrin ligand on blood–brain tumour barrier (BBTB) and GBM cells, and DCDX is a ligand of nAChRs present in the BBB. These liposomes opted for transcytosis pathway for brain capillary endothelial cells via their lysosomal compartments. The extraordinary stability of both peptide ligands in lysosomal homogenate enabled glioma targeting and exocytosis from brain capillary endothelial cells. Cellular uptake assays showed that dual-labelled liposomes have the ability to effectively traverse the BBB and BBTB monolayers, bypass the enzymatic barrier and target 3D tumour spheroids in addition to brain capillary endothelial cells as well as tumour cells. Its targetability towards cerebral glioblastoma was confirmed in vivo with the help of ex vivo imaging and histological tests. When compared to unmodified liposomes and liposomes modified with a single peptide ligand, doxorubicin liposomes modified with both DCDX and c(RGDyK) demonstrated greater anti-glioma activity and increased median life of nude mice with GBM. Therefore, ability of liposome to successfully overcome many barriers and achieve glioma-focused delivery of drug validates its utility in enhancing the treatment efficacy of doxorubicin for glioma. Tamaru et al. [87] examined how a Lep (70–89) acts as a ligand for endothelial cells generated from mouse brain (MBEC4). Liposomes that have been treated with Lep (70–89) had considerably more cellular absorption than unmodified liposomes (PEG-LPs). Lep (70–89) liposomes, according to their theory, were taken up via micropinocytosis; however, it remained unclear if the leptin receptor was directly implicated [87].

### Micelles

Micelles are circular amphiphilic nanostructures with a lipophilic interior and a hydrophilic exterior orientation. Its dynamic structure offers superior kinetic and thermodynamic stability, and it offers many advantages over conventional drug carriers. The lipophilic nucleus carries medication loaded for treatment, while the hydrophilic component stabilizes the micelle in an aqueous system for IV distribution. They exhibit enhanced permeability and retention (EPR) due to their tiny size (less than 50 nm) and hydrophilic shell, which prevents them from being quickly recognized and removed by the reticuloendothelial system. For the active transferrin receptor–based administration of docetaxel (DTX) for the treatment of brain tumours, TPGS micelles were created. Initially, transferrin’s amino ends were covalently attached to acid-functionalized TPGS-COOH. Second, by using the solvent casting (SC) approach, a conjugate (TPGS-Tf) was constructed as micelle’s surface and DTX was entrapped in the lipophilic core of micelle. To demonstrate the effectiveness of docetaxel’s transfer across the BBB, micelles were examined in vivo for brain distribution and their kinetics following intravenous injection. Delivery of DTX via the transferrin receptor can significantly improve the drug’s targeting ability in animals with brain tumours, enhancing therapeutic efficacy while minimizing the adverse effects [88].

Polymeric micelles of Pluronic P105 encapsulated with doxorubicin were developed with aim to facilitate drug accumulation in glioma cells and enhancing BBB transit. The pH-dependent DOX release suggests that the drug releases reasonably quickly under mildly acidic environment and that the transporter state is stable in physiological situations. It was found that glucose transporter and folic acid (FA) receptor–mediated Pluronic P105 polymeric micelles loaded with doxorubicin (GF-DOX) had a better capacity for BBB transportation when it was evaluated in an in vitro BBB model, with a ratio of 21.47% in 4 h. Cells of the C6 glioma internalised the carrier after traversing the BBB model demonstrating the combination effect, targeting activated tumour cells via FA receptor–mediated endocytosis and brain via conveying glucose transporter. A substantial tumour growth suppression ratio and minimal body weight changes were also observed after the intravenous administration of GF-DOX. Therefore, it was determined that the glucose transporter and FA targeting micelles would offer a new treatment for brain tumours [89]. Zhan et al. [90] functionalized a biodegradable PEG-polylactic acid (PLA)-based delivery vehicle with CDX (derivatised peptide) to test the effectiveness of CDX in enhancing nAChR-mediated medication transport via the BBB. For the systemic administration of PEG-PLA, PEG-PLA micelle, a nanocarrier with a hydrophilic shell and a lipophilic core, is frequently employed to carry various hydrophobic medications and imaging probes. Atomic force microscopy reveals that CDX micelle is spherical in shape and has a size of less than 50 nm. Unmodified, CDX-decorated micelles containing the fluorescent dye coumarin 6 were administered into normal nude mice for comparative pharmacokinetic investigations, and then chromatographic analysis of time-dependent coumarin quantities in the blood and brain was performed. Although CDX functionalization of micelles had no minimal effect on coumarin’s pharmacokinetics in the blood, CDX-anchored micelles greatly increased coumarin’s bioavailability in brain, as demonstrated by an elevation of 100% in AUC. These findings indicate that CDX can facilitate drug delivery to the CNS [90].

### Metallic Nanocarriers

A novel class of magnetic resonance contrast agent uses magnetic nanocrystals as its primary component, and these crystals have demonstrated a promising future in initial disorder identification and treatment. Magnetic nanocarrier–based contrast agents still struggle with their capability to pass through the BBB, despite being ideally suited for MRI. Qiao et al. [91] developed various synthetic methods for creating water-soluble, biocompatible nanoparticles containing surface-reactive moieties and superparamagnetic Fe_3_O_4_ nanoparticles. It has also been demonstrated that the resultant Fe_3_O_4_ nanocrystals coated with dicarboxyl-terminated polyethylene glycol can be utilized to create MRI and MRI-SPECT dual-modality molecular markers for in vivo detection of gastric cancer and colorectal cancer. The carboxylated PEG surface provides free surface carboxyl groups for further covalent attachment of bioligands to the particle, giving the nanocrystals biocompatibility [91]. Noninvasive imaging methods have been regarded as crucial clinical tactics for tracking tumour’s early response to therapy. The response in an orthotopic glioma model was analysed with noninvasive MRI and the contrasting material VEGF (121)/rGel, a vascular disrupting agent and RGD peptides conjugated to metal oxide nanoparticles. First, RGD peptides were joined to metallic nanoparticles that were covered in an amphiphilic triblock copolymer that had been cross-linked with PEG. According to in vitro binding experiments, the primary determinant of cellular absorption of particles was the interaction amongst integrin v3 and RGD in HUVECs. In a GBM model, the enhanced targeting of IONP-RGD was seen. Lastly, employing prepared nanoparticles as an imaging contrast agent is a method that is superior to conventional anatomical approaches based on tumour growth measures. The tumour response to treatment at early phases of therapy resulted in lowering of tumour size [92].

### Nanoemulsion

A nanoemulsion (NE) is a very fine oil-in-water or water-in-oil dispersion stabilized by an interfacial layer of emulsifier molecules with sizes ranging from 20 to 600 nm. There are three types of nanoemulsion: (a) oil in water (o/w), (b) water in oil (w/o) and (c) bi-continuous [93]. To enhance the administration of docetaxel to specific tumours, Afzal et al. [94] created a transferrin-coupled docetaxel lipid nanoemulsion. By using a homogenization and ultrasonication method, the basic lipid nanoemulsion was created (FP). Using the EDC process, lipid nanoemulsion (FS) globules that contain stearylamine were tagged with transferrin. It was observed that the transferrin-coupled lipid nanoemulsion had small globule size, encapsulation efficiency and delayed release. Moreover, the imaging study findings in tumour-induced mouse model showed that FT had a substantial targeting capacity by 3.54-fold in comparison to control FP without Tf ligand and 2.62-fold compared to FS. The increased anticancer activity of the FT in comparison to control NEs was also confirmed by the pharmacodynamic studies. Thus, the transferrin delivery system (FT) has the ability to be formulated into a newer, improved cancer treatment method. To determine the effect of the FT delivery method, it must be tested on cancer patients [94].

### Polymeric Nanoparticles

Polymeric NPs (PNPs) have played a very important role in modern medicine, from contrast agents in medical imaging to nanocarriers for delivering genes to certain cells. NPs are smaller than bulk materials, which make them different from those in a variety of respects, such as energy absorption, chemical reactivity and biological mobility. PNPs are particles less than 1000 nm in size. They are made of natural and synthetic polymers, including albumin, collagen and chitosan, which are primarily being studied for use in biomedical applications. Synthetic polymers include PLA and PLGA [95]. PNPs are the most adaptable and well-liked nanocarrier vehicles for drug delivery to the CNS because they can successfully transport pharmaceuticals across the blood–brain barrier when functionalized with appropriate cell-penetrating peptides and/or targeting ligands [96].

Bovine serum albumin NPs conjugated to glutathione were developed as a newer biodegradable delivery system for the brain. Both in vitro and in vivo transport capabilities were assessed. Using the carbodiimide chemistry and EDC as a mediator, BSA NPs were activated and coupled to the amine functionalities of glutathione. Ellman’s assay suggested that each BSA nanoparticles had about 750 conjugated units of glutathione on the BSA nanoparticles’ surface. Fluorescein sodium was employed as a representative hydrophilic chemical to assess the glutathione-conjugated BSA nanoparticles’ brain transport abilities. Developmental compounds were tested for MDCK-MDR-1 endothelial and glial cells for penetration and neuronal uptake, respectively. The single layer of MDCK-MDR-1 endothelial tight junction was considerably more permeable to glutathione-conjugated BSA NPs than to nonconjugated NPs and fluorescein sodium solution. In a similar manner, glutathione-conjugated NPs demonstrated greater absorption by glial cells in comparison to nonconjugated nanoparticles and fluorescein sodium solution, as per microscopic studies. Glutathione-conjugated NPs transported about three times more fluorescein sodium to the rat brain after intravenous injection than unconjugated NPs. Substantial in vivo and in vitro research points to glutathione-conjugated BSA nanoparticles as a low-toxicity brain-medication delivery method [97]. Another study validated the potential of polymersomes made of diblock copolymers functionalized with a particular antibody for medication localisation to the brain. As a model system, the human insulin receptor (IR) on brain endothelial cells was employed to execute an active and physiological targeting technique. Moreover, in comparison to an anti-transferrin receptor antibody, monoclonal antibody against the IR has a nearly tenfold greater BBB transit. Hence, to transport medications via the BBB, targeting insulin receptor is a viable strategy [98].

Another research sought to create a biodegradable nanoparticles that could carry medications across the BBB and had suitable surface changes. Initially, PLGA NPs were developed with the nanoprecipitation technique. Next, trimethylated chitosan (TMC) was bound covalently to the PLGA NP surface. Using 6-coumarin as a probe, the intravenous transport of PLGA NPs was contrasted with the brain reception of TMC/PLGA NPs following intravenous administration under microscope. The cytotoxicity of the nanoparticles was further examined using the MTT test. The neuroprotective effects of several coenzyme Q10 (CoQ10) formulations were next assessed in an APP/PS transgenic mouse during a water maze test. CoQ10 was selected as the model medication. Standard immunohistochemistry procedures were used to stain the brain sections, and the biochemical elements related to oxidative stress were investigated [99]. Another study uses HSA nanoparticles with a specific anti-insulin receptor antibody or insulin linked to it using the NHS-PEG-MAL-5000 crosslinker. As IR is an especially compelling target for the nanoparticle-assisted drug delivery to the brain, the development of HSA NPs with covalently linked insulin and an antibody against this receptor has shown promise for brain drug delivery [28].

For patients with brain tumours undergoing surgery or receiving postoperative radiography, a particular MRI contrast agent is essential. In order to identify gliomas in vivo, lactoferrin-linked metallic nanoparticles (Lf-SPIONs) were investigated. The Lf-SPIONs’ T2 relaxivity of 75.6 mM/s, with size less than 100 nm and saturation magnetization of 51 emu/g Fe, suggested its suitability for MRI. Lf-SPIONs were found to be more sensitive than SPIONs at depicting brain glioma on MR images when used with a C6 glioma rat model. Lf-SPIONs were used in vivo to capture significantly increased T2-weighted pictures of brain gliomas up to 48 h after injection. Furthermore, after 48 h, Lf-SPIONs could clearly be seen in the vascular area of the tumour slices. Using molecular studies, high levels of Lf receptor expression in comparison to normal brain tissues were demonstrated in brain tumour tissues. Results revealed the possible use of Lf-SPIONs as a precise and focused MRI contrast material for detection of gliomas [100].

In an experiment, cationic polymers (chitosan (CS) or polyethyleneimine (PEI)) were modified with stearic acid (SA) and Lf and their penetration via the BBB was determined. Both the cellular uptake by nerve cell types and the efficacy of recombinant pre-miR-29b at silencing genes were investigated in vitro. After a 1-h transfection, tagged SA-Lf systems revealed high fluorescence in the cytoplasm and nucleus of RBE4 cells, demonstrating pre-miR-29b transport to nerve cells. Experiments on the recombinant pre-miR-29b delivery over the BBB revealed that the formulation transmitted more than 50% of the gene in 4 h, compared to PEI-SA-42% Lf’s in that particular time period. Hence, a newer method for the dual targeting of delivery system is revealed, opening up fresh possibilities for the administration of nanomedicines. This method utilizes a novel drug delivery system that benefits from special characteristics of the several immobilised ligands [101]. To deliver etoposide via the BBB and therapy of malignant glioblastoma of the human brain, Lf and FA were cross-linked on PLGA NPs. The NPs penetrating the HBMECs modulated by astrocytes also inhibited the proliferation of glial cells. The etoposide entrapment efficiency and sustained drug release characteristics of NPs were acceptable. When etoposide was delivered through the BBB using these NPs as opposed to PLGA NPs, the permeability coefficient rose by around three times. These NPs were the most effective antiproliferative agents against the development of glial cells, followed by folic acid/PLGA NPs, free etoposide solution and PLGA NPs. Moreover, Lf receptor immunostaining on HBMECs and folate receptor immunostaining on glial cells at the time of endocytosis demonstrated the targeting ability of these NPs. Hence, it was concluded that Lf/FA/PLGA NPs with encapsulated etoposide may be a useful chemotherapeutic medication to increase the etoposide transport to malignant brain tumours during in vivo studies [102].

For targeting transferrin-mediated receptor transcytosis, overexpressed in brain tumour, Kang et al. [103] developed many peptide-gene delivery systems with the T7 chain extension and next examined gene delivery ability to convey genes. The physical characteristics of peptide vectors or peptide/DNA complexes were also tested. The effectiveness of in vitro transfection was examined in both normal and glioblastoma cells. Amongst these, the PT-02/DNA combination had the best insertion efficiency in glioblastoma cells and the least amount of cytotoxicity in normal cell lines. It was also capable of moving DNA via the BBB model. The developed peptide carriers provide a possibly successful approach for glioma gene delivery [103].

In order to transfer protein to the brain, a CS-linked Pluronic nanocarrier containing the particular target peptide (rabies virus glycoprotein; RVG29) was used. Using Cy5.5 conjugation to the nanocarrier, the in vivo brain deposition of the nanocarrier in mice after IV administration was optically observed. The observations demonstrated that the Pluronic nanoparticles conjugated with both chitosan and peptide was effective for the concentration in brain tissue and was noticeably superior to the nanoparticles linked only with peptide. By nanoparticles loading and monitoring its biodistribution, galactosidase was efficiently transported and deposited in brain. The given protein kept its bioactivity in the brain as well. Hence, these nanocarriers may be helpful in the detection and treatment of brain disorders [104]. Another work examines the impact of zidovudine (ZDV) to gain access within brain using CRM197-grafted poly-cyanoacrylate NPs. ZDV was loaded onto cyanoacrylate NPs to allow it to penetrate the HBMEC cell line. Conjugated nanoparticle showed improved penetration coefficient of ZDV through the BBB and higher loading efficiency in drug carrier. A greater amount of CRM197 was grafted, which improved both the amount of ZDV-loading and the permeability of ZDV across endothelial junction. These NPs have the potential to be effective brain-specific carriers for the delivery of ZDV [105]. DGL NPs were prepared encapsulating plasmid DNA and decorated with Lep 30 to produce DGL-PEG-leptin 30. Brain capillary endothelial cells containing leptin substrates were studied to learn more about the cellular uptake characteristics and mechanism. Moreover, leptin receptor–expressing brain parenchyma microglial cells like BV-2 cells may encage ligand-receptor-mediated endocytosis, which would improve the efficiency of leptin-conjugated NPs to transfect genes. The prepared NPs were demonstrated to cross the BBB model efficiently and to deposit more in the brain following intravenous route, leading to a comparatively high efficiency of gene transfection. In addition to cellularisation, the NPs showed lowered toxicity. Therefore, DGL-PEG-leptin 30 offers a secure and nonintrusive technique for delivering a gene across the BBB for brain-targeted delivery [17].

### Solid Lipid Nanoparticles and Nanostructured Lipid Carriers

The advantages of SLNs over polymeric NPs are due to their lower cytotoxicity, higher drug loading and scalability. SLNs have shown great potential for brain targeting and have been widely studied for CNS delivery [106].

In hCMEC/D3 cell monolayers, apolipoprotein E–functionalized SLNs were examined. These nanosystems are excellent for brain administration due to their average diameter of > 150 nm, lipophilic properties and negative charge on the surface. In comparison to the nonfunctionalized SLNs, SLN-palmitate-ApoE and SLN-DSPE-ApoE increased cellular uptake by 1.8- and 1.9-fold, respectively, in microscopic studies. Nanoparticles were found to be able to penetrate the BBB primarily through a transcellular pathway, with clathrin-based uptake being identified as the preferred process involved in cellular internalisation. Understanding the processes by which these nanosystems traverse the BBB may enhance their use in the drug transport to the brain [107] delivery approach for donepezil to circumvent BBB more effectively.

Zwain et al. [108, 109] developed functional nanostructured lipid carriers (NLCs) of DTX and tailored their composition to enhance BBB and BBTB permeation and target the GBM cells. The authors demonstrated that combination of four liquid lipids at low concentration along with solid lipid improved the BBB permeability when evaluated in an in vitro BBB model. The DTX-NLCs showed high cellular internalisation in GBM cells as well as low IC50 [110]. In another recent study, authors conjugated GLA or alpha-linolenic acid (ALA) to DTX-NLCs and demonstrated that polyunsaturated fatty acid (PUFA)-functionalised NLCs enhanced the GBM cell internalisation as compared to bare DTX-NLCs and positively impacted the in vitro tumouricidal activity of CNS negative anticancer drug DTX [109].

### Nanotheranostics for BBB-directed Delivery

Theranostic NPs have recently shown great promise and efficiency in providing targeted drug administration because of their special physiochemical characteristics, which include their nanoscale size, drug encapsulation, adsorption or combination of contrast agents at their exterior. With the use of a single formulation of biocompatible and biodegradable nanoparticles, these multifunctional nanosystems coated with targeted agents have the potential to be used for disease detection, imaging and treatment. At the moment, a number of theranostic nanoplatform types are being investigated for the diagnosis and therapy of neurological disorders, including lipid NPs, PNPs, inorganic NPs and others [111–115] Because it is nontoxic, biocompatible and biodegradable, chitosan is the most often utilized natural polymer as a theranostic agent for neurological illnesses. Because synthetic polymers like PLA and PLGA are biocompatible and biodegradable and have low immunogenicity, they are frequently used, just like natural polymers, to create theranostic medicines for neurological illnesses [112]. Moreover, the most often employed theranostic nanostructures are inorganic NPs including iron oxide, ceria, gold and quantum dots because of their multifunctionality, facile surface modification, controlled size and excellent biocompatibility. However, the possible toxicity and instability of these systems limit their clinical uses. Furthermore, a number of researchers have shown a considerable interest in nanosystems such as dendrimers, carbon nanotubes (CNTs), and ultra-compact nanoparticles NPs (UCNPs) in recent times. Because of a nonlinear optical process, UCNPs have the unique ability to produce high-energy visible radiations from low-energy near-infrared (NIR) radiations. It is interesting to note that UCNPS are activated within a narrow absorption band at approximately 975 nm, which falls within the near-infrared range that tissues are thought to have as their “optical transparency window” (700–1100 nm). Biological tissues have been found to exhibit less autofluorescence and comparatively little scattering and absorption at 975 nm while collecting the NIR UC emission. Therefore, the surrounding biological tissues are not photodamaged when UCNPs are excited at this wavelength. Hence, UCNPs possessing notable optical characteristics as broad Stokes shifts, slender emission peaks and strong chemical and physical stabilities can serve as effective intelligent nanoprobes for prospective nanotheranostic uses [113, 114]. Dendrimers with distinct functional groups have a well-organized three-dimensional structure and can easily modify their surface, which gives them the ability to load large amounts of drugs and makes them useful as imaging and diagnostic agents. Additionally, surface-modified dendrimers show improved biocompatibility and decreased cytotoxicity for in vivo applications. Similarly, CNTs have special mechanical and electronic attributes appropriate for use in biomedical applications. Surface modification and functionalization of CNTs with hydrophilic or biocompatible substances enhance their target-specific delivery to tissues or cells, water dispersibility and least toxicity. Comparing CNTs to other theranostic NPs, it is interesting to note that the intrinsic NIR photoluminescence optical and thermal properties of CNTs have demonstrated considerable promise for brain-targeted theranostic applications [115, 116].

Different varieties of lipid NPs such as liposomes, solid lipid NPs, porphysomes and lipid-coated calcium phosphate are available based on the types of lipid and their physiochemical properties [110]. These NPs can deliver substances that are both hydrophilic and hydrophobic. Additionally, their surface can be altered or functionalized with different molecules for a range of uses, including ligands as a noninvasive biodistribution monitoring tool or imaging agent. By functionalizing with different ligands, such as peptides, antibodies, aptamers and others, which bind to the expressed receptors on the surface of brain endothelial cells and allow these NPs to penetrate the blood–brain barrier, active targeting of these NPs can be accomplished [117]. Similarly, hydrophilic polymers including PEG, poly(acryloylmorpholine), poly-*N*-vinylpyrrolidones, polyvinyl alcohol and poly[*N*-(2-hydroxypropyl)methacrylamide] can be applied to the surface of NPs for passive targeting. It is interesting to note that these polymers prevent macrophage and monocyte identification, lowering the possibility of opsonization and delaying the removal of NPs from the bloodstream [118].

## Concerns Associated with Application of Nanocarriers for Brain Targeting

The overall drug targetability is dependent on various factors like the mainly inherent nature of medicament, affinity for brain organelles, stability within intracerebral conditions, metabolism pathway and circulation half-life along with safety associated with the drug. Human brain is well equipped to keep all the foreign pathogens such as microbes, parasites and toxic materials away from the CNS. The newer CNS drug transport approaches increase the overall chances of letting other neurotoxins gain access within brain compartment. Such systems mainly concentrated on delivery maximum fraction of nanocarriers across the BBB that increases the nanocarrier concentration within brain compartment. Such higher accumulation of drug may cause untoward challenges associated with neurotoxicity. Premature drug release can also be a major concern while developing brain-targeted systems. Such early dissemination of drug from nanocarriers leads to reduction of the neuroprotection, enzymatic degradation, development of toxic byproduct or untoward drug delivery. Such challenges have raised the concern associated with safety of nanocarriers. Few drugs are also reported with the regaining access within the systemic blood flow and get metabolized quickly. All the probable outcomes hamper the overall efficiency of carrier-based targetability. Therefore, an ideal carrier should protect the drug within the core till it reaches to the target site. For such site-specific delivery, various approaches like pH-sensitive drug delivery, ligand-specified system and externally triggered responsive nanocarriers were used [119].

### Surface Characteristics of Nanocarrier

Nanocarriers are made of synthetic polymers, cross-linkers, surfactants and stabilizers. These excipients have impact on physicochemical properties of prepared nanocarriers such as charge, particle size, solubility stability, ligand binding efficiency and lipophilic-hydrophilic ratio. These characteristics made the efficient delivery of carriers across the BBB. Ideally, particle size below the size of 150 nm successfully traverses the BBB portal. Surface charge density is also vital drug permeation across the BBB. Hydrophilic molecules gain access within brain via diffusion. Ligand availability and stability within carrier is also important for ligand-specific targeting [120].

### Stability of Protein Backbone–containing Nanoparticles

Various nanomaterials are comprised protein backbone, assembled in a stable form. But once administered under physiological conditions, protein undergoes conformational destabilization, allowing free exchange within the brain compartments. Protein molecules give rise to the protein-based corona within suitable physiological conditions. Presence of such corona may lead to delayed circulation interval and compromise interactive ability of nanosystems via camouflaging the receptor and ligand. Few studies have highlighted the application of proteins in brain drug targeting efficiency.

### Nontargeted Delivery of Nanocarriers

The drug targetability of carrier is dependent upon the interrelation with receptor and substrate. Such carrier and substrate expression is crucial for targeting ability. Receptors are scattered in the body at various locations in low to highly overexpressed manner. Such heterogenicity can cause nonselective drug release at different sides and can cause substantial untoward effect [121].

## Clinical Status of Nanomedicines for BBB Penetration

Many clinical studies are being conducted over the past two decades in an effort to study the extent of nanodelivery devices for brain cancer therapy. Drug- or siRNA-loaded nanomedicines have been the main focus of the many of these phase I and phase II research studies. The primary objective of these clinical interventions is to determine and evaluate the efficacy and safety of nanosystem [122].

Under one such phase II clinical intervention, glioblastoma patients treated with PEG-coated-liposomal DOX (PEG-DOX; Caelyx™) along with prolonged administration of temozolomide and with radiation revealed 30.2% progression-free survival (PFS) rate after 12 months of regimen administration. The result suggested that the continuous administration of temozolomide, PEG-DOX and radiation does not induce toxic effect. Yet, the inclusion of PEG-DOX and continued delivery of temozolomide did not show any significant disagreement between previously reported data [123]. A phase I clinical intervention examined in patients suffering from multiple brain metastases the intravenous efficacy, tolerability and side effects of gadolinium-based nanoparticle AGuIX in combination with conventional whole-brain radiation [124]. Under similar clinical intervention (phase I study), gadolinium metal nanoparticles were administered to amplify the effect of radiation in 15 volunteers with four different types of brain brain metastases (melanoma, lung, colon and breast). These nanocarriers were found to aggravate within the region of metastasis and enhance the bioimaging contrasting ability as compared to clinical contrasting agent. The nanocarriers were observable within metastases till 1 week post administration. Furthermore, radiosenstising properties of nanoparticles were utilised  by administering the nanoparticles to patients prior to radiotherapy. This protocol has been extended to a multicentric phase 2 clinical trial including 100 patients [125]. The preclinical studies certain times show promising results of nanomedicines within preclinical models, whereas these nanomedicines often do not yield similar therapeutic effect when studied in a clinical trial. For instance, in a phase I/II clinical research, ITV DepoCyt and Ara-C-temozolomide liposome were used to study the suppression of the progenitor glial cells around the ventricular system with progressed-stage brain tumours. The study was terminated due to insufficient patient participation, inadequate Karnofsky Performance Scale (KPS) (which assesses functional impairment of patient upon illness reappearance) and the recent accessibility of FDA-approved medications, such as Avastin (bevacizumab), after the study’s inception (https://www.clinicaltrials.gov/, NCT01044966, September 2009). Studies on drug entrapment, targeting effectiveness, particle shape and size, release kinetics and the establishment of successful protocol must also be conducted in addition to biodistribution, efficacy and safety studies. To evaluate nanotoxicology throughout preclinical and early clinical testing, sensitive and standardizable assays for cell line analysis and animal study approaches are required [126]. It is necessary to provide proof for a nanodelivery system’s capacity to treat gliomas even after there is substantial support for its security and effectiveness. The development of nanomedicines for commercial use is difficult due to challenges in clinical trials. Because of their structure, most nanoparticles are challenging to manufacture with respect to quality control and reproducibility. Despite research and development and production, there are still many barriers to commercializing nanomedicines, including intellectual property rights, governmental restrictions and general cost-effectiveness in comparison to existing treatments. Various FDA-approved marketed drugs are shown in Table 5 [127]. Currently, few clinical interventions dedicated to brain targeting via receptor-based voyage are undergoing. Under one such ongoing study, the complexity of migraine pathophysiology, focusing on the challenging aspect of headache generation, is addressed. While the traditional vascular hypothesis emphasizes cranial artery dilatation, this investigation considers the potential involvement of perivascular pain-sensitive structures, drawing parallels from conditions like giant cell arteritis. The study hypothesizes an association between unilateral migraine without aura and ipsilateral inflammation of cranial arteries and meninges, proposing that sumatriptan may inhibit this perivascular inflammation. To explore these hypotheses, clinicians employ MRI techniques, utilizing ultrasmall superparamagnetic iron oxide (USPIO)-MRI and blood–brain barrier imaging (BBI)-MRI to visualize perivascular inflammation. Cilostazol, a phosphodiesterase 3 inhibitor, is used to pharmacologically induce migraine headaches in study subjects. This research aims to provide insights into the mechanisms underlying migraine headaches, offering potential avenues for therapeutic intervention, with a particular focus on the role of perivascular inflammation and the efficacy of sumatriptan in mitigating its impact.

**Table 5:** FDA-approved marketed products for brain targeting delivery [128]

S.No	Product	API	Indication/disease	Key points
1	Austedo	Deutetrabenazine	Huntington’s disease	Treats the involuntary movements (chorea)
2	Ocrevus	Ocrelizumab	Multiple sclerosis	Reduces the number of circulating immature and adult B lymphocytes while protecting CD20-negative plasma cells
3	Radicava	Edaravone	Amyotrophic lateral sclerosis (ALS)	Reducing the impact oxidative stress
4	Nuplazid	Pimavanserin	Hallucinations and delusions associated with psychosis	Acting on serotonin 2A receptor as an inverse agonist as well as antagonist
5	Rexulti	Brexpiprazole	Schizophrenia	Partial agonism on some serotonin and dopamine receptors, while concurrently antagonising other serotonin receptors
6	Vizamyl	Flutemetamol F-18 injection	Radioactive diagnostic drug for Alzheimer’s disease	Flutemetamol (18F) binds to -amyloid neuritic plaques
7	Tecfidera	Dimethyl fumarate	Relapse of multiple sclerosis	Lessens the inflammation brought on by the immune system attacking myelin, minimizing myelin damage
8	Brintellix	Vortioxetine	Major depressive disorder	Strong affinity for many serotoninergic receptors and inhibition of the serotonin transporter (SERT)
9	Dotarem	Gadoterate meglumine	MRI-based brain imaging	Gadolinium-based contrast agent
10	Ingrezza	Valbenazine	Tardive dyskinesia	Reversible inhibitor of VMAT2
11	Xadago	Safinamide	Parkinson’s disease	MAO-B inhibitor
12	Zinbryta	Daclizumab	Multiple sclerosis	Modifies the interleukin-2 (IL-2) signalling without resulting in any reduction of immune cells
13	Spinraza	Nusinersen	Spinal muscular atrophy (SMA)	Contains an antisense oligonucleotide (ASO), which regulates the mutations induced in chromosome 5q
14	Vraylar	Cariprazine	Schizophrenia and bipolar disorder	Depending on the amount of dopamine D2 and serotonin 5-HT1A present in the brain, behaves as either an agonist or an antagonist
15	Aristada	Aripiprazole lauroxil	Schizophrenia	5-HT2A receptor antagonist activity and partial agonist activity at D2 and 5-HT1A receptors
16	Plegridy	Peginterferon beta-1a	Relapse of multiple sclerosis	PEGylation of interferon beta-1a
17	Aptiom	Eslicarbazepine acetate	Epilepsy-associated seizures	Reduces the pain and seizure-inducing nerve impulses

These are FDA-approved API molecules for the treatment of neurodegenerative disorders

Another ongoing study aims to investigate the potential synergistic and safe effects of a combination therapy involving probenecid and *N*-acetylcysteine (NAC) in preserving GSH levels and reducing oxidative stress in children suffering from severe traumatic brain injury (TBI). A total of 20 participants aged 2 to less than 18 years are enrolled in a randomized, controlled trial. The primary objectives are to assess the safety of administering this combined treatment and to evaluate its impact on antioxidant reserves in both CSF and serum. The regimen involves administering probenecid at the same dose used as an adjunct to antibiotic therapy and NAC at the same dose employed for acetaminophen-induced liver disease over a 3-day period. A control group receives vehicle treatments. Key endpoints include the safety profile of drug administration and the measurement of antioxidant reserve (AOR) levels in CSF and serum. The study is grounded in the hypothesis that maintaining antioxidant levels within the brain may confer neuroprotective benefits. Secondary outcomes encompass examining CSF and serum concentrations of probenecid, NAC, GSH and phenytoin. Adverse events occurring during drug administration post-traumatic brain injury are closely monitored by a local Data and Safety Monitoring Board. This ongoing scientific inquiry seeks to provide valuable insights into the safety and efficacy of the proposed combination therapy in paediatric patients with severe traumatic brain injury [129, 130]. In another intervention, clinicians focused on brain asphyxia and targetability. During the perinatal period, certain infants encounter a critical condition known as asphyxia, characterized by insufficient blood and/or oxygen supply to the brain and other vital organs. This life-threatening circumstance contributes to nearly one-fourth of all global neonatal deaths, frequently resulting in profound consequences such as severe brain damage, cerebral palsy, epilepsy, and cognitive impairments in daily functioning. Presently, there exists no established therapeutic intervention to ameliorate the cerebral damage induced by perinatal asphyxia. Notably, sildenafil, a pharmaceutical agent commonly administered to infants with pulmonary hypertension, exhibits potential for addressing this unmet medical need. Recent investigations utilizing a laboratory model simulating birth asphyxia suggest that sildenafil holds promise in mitigating the cerebral repercussions associated with this condition. Furthermore, preliminary clinical studies have demonstrated the feasibility and safety of administering sildenafil to human neonates affected by perinatal asphyxia. These clinical endeavours have yielded encouraging indications that sildenafil may enhance cognitive functioning, aligning with the insights gleaned from experimental models. Building upon these preceding investigations, research endeavours aim to ascertain the reparative potential of sildenafil in attenuating neonatal brain damage resulting from asphyxia. This comprehensive investigation seeks to assess the safety and efficacy of sildenafil administration in a substantial cohort of neonates impacted by birth asphyxia, focusing on neurological and cardiopulmonary outcomes. This project holds the promise of elucidating whether sildenafil emerges as a viable therapeutic modality for ameliorating cerebral damage in neonates affected by perinatal asphyxia. Additionally, the outcomes of this research may pave the way for innovative strategies to enhance the future quality of life for these vulnerable neonates (https://www.clinicaltrials.gov/, NCT02372409, August 14, 2015).

## Challenges Interlinked with BBB-directed Nanocarriers

The preceding decades have witnessed noteworthy advancements in clinical CNS therapy; nevertheless, it remains a widely acknowledged reality that these sophisticated strategies have failed to yield significant alterations in the mortality rates of brain targeting or improvements in the overall quality of life for afflicted patients. Although nanocarrier-based drug delivery systems present a promising avenue, numerous unresolved issues still persist. A paramount concern pertains to the potential toxicity associated with nanodrug formulations. The exceedingly diminutive size of these carriers raises the prospect of tissue accumulation. Specifically, metallic NPs containing elements such as iron, cobalt, zinc, gold or other heavy metals may manifest tendencies to accumulate in vital organs such as the liver, lungs or brain, giving rise to chronic toxic effects. Metallic NPs have the capacity to generate free oxygen species, thereby contributing to potential toxicity. Moreover, their nonbiodegradable nature implies a protracted environmental presence, resulting in sustained human exposure with uncertain repercussions for the ecosystem. Reports suggest that CNTs may induce the formation of reactive oxygen species, potentially precipitating lipid peroxidation, mitochondrial dysfunction or cellular damage. Addressing these concerns is imperative for the continued development and application of nanodrug formulations in the domain of clinical neurotherapeutics. Practically, a multitude of potential toxic effects associated with nanodrug carriers exist, many of which remain unidentified. The manifestation of these toxic effects is contingent upon various factors, including size, shape, chemical composition, biocompatibility, route of administration and degradation mechanism, necessitating comprehensive investigation. Subsequent to administration, uncertainties persist regarding alterations in the properties of nanomaterials within brain microenvironments and their impact on complement activation, blood coagulation and overall human immunity. Consequently, a thorough investigation of these critical factors pertaining to the in vivo behaviour of nanodrug carriers and their influence on healthy brain cells is imperative [131].

In the case of neurotherapeutic drug–loaded nanocarriers, the determination of appropriate dose ranges is of paramount importance. In vivo experiments elucidating both blood and brain pharmacokinetics of nanomaterials are essential to comprehend their absorption, distribution, metabolism and excretion (ADME) behaviours. Nevertheless, a substantial dearth of comprehensive preclinical data on nanodrug carriers for brain delivery persists, compounded by a scarcity of information regarding in vitro-in vivo correlation. Consequently, drawing conclusive outcomes regarding the efficacy of such nanodrug carriers for brain tumour treatment remains challenging. Despite the abundant reports and studies on nanodrug formulations, only a scant proportion of these nanosystems (< 10%) have successfully obtained regulatory approval for market entry. The inadequacy of in vitro-in vivo correlation for nanodrug carriers poses a significant hurdle to regulatory clearance. Additionally, critical aspects such as cellular interactions, tissue transportation, diffusion and biocompatibility remain systematically unexplored, lacking comprehensive investigation employing appropriate animal models. Concerns regarding the practicality and rationality of in vivo experiments on laboratory animals have been raised by medical experts, given the considerable anatomical and physiological differences between laboratory animals and humans. It is noteworthy that laboratory animals, particularly rodents, do not frequently develop brain tumours or neurodegeneration as observed in humans. Moreover, variations in reactions and metabolism between laboratory animals and human subjects are evident, wherein a substance deemed nontoxic to animals may exhibit toxicity to humans, and vice versa. The absence of precise knowledge regarding the mechanisms underlying the development and progression of neuronal damage and neurodegeneration, along with the identification of biochemical factors, specific antigens or proteins involved in these processes, complicates their establishment in experimental animal models. Consequently, the utility of genetically modified animal models for testing the in vivo efficacy of nanodrug carriers, as well as the reliability of data generated from such animal models, remains a subject of contention amongst scientists [132, 133].

In the practical domain of large-scale manufacturing for nanodrug carriers, numerous unresolved challenges persist. Despite the inherent advantages, pharmaceutical companies exhibit reluctance to make substantial investments in nanocarrier-based drug delivery platforms. Consequently, a substantial portion of research outcomes is confined to academic or small-scale research laboratories, failing to transition effectively from bench to bedside. Amongst various factors contributing to this scenario, a prominent concern is the manufacturing cost. Typically, the overall production cost of nearly all nanocarrier-based formulations intended for human applications exceeds that of conventional formulations. Additionally, practical issues during the production phase, such as batch-to-batch variation, low drug loading capacity, and stability concerns, further dissuade pharmaceutical companies from undertaking associated risks [134].

### Nanotoxicological Concerns

While nanocarriers represent a potent tool for traversing the BBB, several formidable challenges necessitate resolution. Firstly, following substantial absorption of nanocarriers into the brain, the precise distribution within the cerebral organ remains inadequately elucidated in numerous studies, posing potential risks to this intricate organ. Secondly, the metabolism of nanocarriers assumes paramount importance. On the one hand, a substantial proportion of nanocarriers comprises inorganic materials, including gold nanoparticles, iron nanoparticles, cerium oxide nanoparticles, molybdenum nanoparticles and silica nanoparticles, characterized by limited metabolic pathways, potentially leading to their accumulation in the brain. These materials may contribute to neurodegeneration through mechanisms involving the induction of mitochondrial dysfunction, redox imbalance, apoptosis, autophagy, impaired lysosomal activity, cytoskeletal damage, vesicle trafficking perturbations, neuroinflammation and microglial activation. Addressing these issues is imperative for the safe and effective utilization of nanocarriers in brain-targeted drug delivery [135].

### Nanocarrier-Based Hindrance in Targetability and Safety

Various nanocarriers have explored for its brain-directed targeting, cargo loading and site-specific delivery. Amongst all these carriers, nanoemulsion is most widely utilized owing to its simple fabrication procedure and ease of preparation. Despite such advantages, clinical and preclinical applications of nanoemulsion are compromised mainly due to surfactant-mediated toxicity, mucociliary damage and early clearance rate. Additionally, nanoemulsion surface orientation does not allow enough sites for conjugation of site-directed peptide or ligands. Hence, newer approaches for improving the nanoemulsion-based targetability are needed [136]. In terms of quantum dots (QDs), bioconjugation introduces challenges in achieving effective delivery to target cells. The aggregation of QDs can result in cytotoxicity, leading to cellular death. Certain materials employed in the preparation of QDs may contribute to neurotoxic effects. The metabolism and excretion pathways of QDs remain unclear, posing potential toxicity concerns within the body. Coating the core material with a shell presents difficulties in maintaining precise control over the size of the QDs. Additionally, the blinking property and surface characteristics of quantum dots contribute to their deterioration. Addressing these issues is crucial for advancing the application of QDs in biomedical contexts with enhanced safety and efficacy for CNS targeting [137]. Inorganic and organic nanoparticles are widely utilized for preclinical and clinical evaluation of neurological ailments showing that inherent toxicity, low encapsulation efficiency (hydrophilic therapeutics), poor solubility, prompt degradation, difficulty in industrial scale-up and use of organic solvents during fabrication process have reduced the pharmaceutical applicability of these carriers. Whereas lipidic nanocarriers are reported with low stability, faster clearance, scalability issues which reduced transfection (due to high affinity of the nucleic acid with the lipid) and poor loading capacity for hydrophilic moieties have reduced its applicability in BBB targeting [138]. The cytotoxicity of dendrimers is primarily contingent upon factors such as generation, concentration, incubation duration and the nature of terminal groups present on their surface. Thus far, surface modification has been recognized as a judicious design strategy to regulate cellular interactions and mitigate the neurotoxic potential of dendrimer-based biopolymers. Beyond their potential neurotoxic effects, the swift systemic elimination of dendritic macromolecules hinders their utility in drug delivery. Notably, smaller dendritic macromolecules (G2–G4) can undergo rapid renal filtration owing to their diminutive size, while larger dendrimers are predisposed to recognition and clearance by the reticuloendothelial system. Notably, PAMAM and PPI dendrimers exhibit expeditious clearance from the bloodstream via the mononuclear phagocyte system, resulting in a substantial accumulation of administered dendrimers in the liver, kidney and spleen; contingent upon dendrimer generation; and surface characteristics. To address these challenges, surface functionalization with diverse neutral entities, such as PEG chains, is being explored [139].

## Future Perspective

The development of neurotherapeutics for amelioration of neurological sequelae is a herculean task as it involves BBB hindrance and inadequate absorption of therapeutic moiety. The BBB is an essential biological barrier that selectively allows essential molecules like glucose, amino acid, exchangeable gas and water molecules. Various neurodegenerating diseases are interlinked with BBB integrity, and scientists are paving their way to exploit BBB’s inherent selective nature for essential therapeutics transportation. Although many neurological challenges are interlinked with the destruction in structural orientation of the BBB such as Parkinson’s disease, multiple sclerosis, Alzheimer’s disease, brain cancers, ischaemic stroke, hypoxia, epilepsy, cerebral palsy and autistic spectrum disorder. Hence, designing strategies to circumvent sturdy BBB restriction and explore the other way for ensuring successful brain targeting can be a new virtue in neurotherapeutics [140].

Nanotherapeutics is a latest and safe imperative tool for newer drug delivery strategy accompanied with protein, polymers and peptides. It is a futuristic tool for safe transmission of neurotherapeutics without altering the physiological conditions that provides prolonged residence time, better stability, improved bioavailability and target-specific delivery. Nanotechnology employed administration via invasive and noninvasive methods, and both methods offer advantages along with few challenges. Biogenic excipients such as protein and self-degradable polymers are widely utilized owing to their safety and biodegradability. Although with all the advancement, nanotechnological carriers are reported to give rise to neurotoxicity upon prolong administration. Therefore, selection and concentration of excipients is essential for safety and targetability via nanotechnological assistance [141].

## Conclusion

The treatment efficiency for various neurological diseases is challenged due selective restriction imposed by the BBB. In the present review, we have described basic framework of the BBB, developmental stages of the BBB, transportation mechanisms across the BBB, dyshomeostasis of the BBB, its impact on development of neurological sequelae, nanotechnological assistance and current available platform for targeting. We have emphasized more on receptor-based targeting as it promises enhanced targeting efficiency. Nanotechnological platforms have accelerated the targeting via modulating biochemical pathways, and these nanocarriers successfully target via both invasive and noninvasive route for brain delivery. In the future, incorporation of gene-based therapeutics encapsulated in nanocarriers can further improve the lacunae in brain targeting via BBB modulating strategies. A more tailored precision-based therapeutics system will be boon for the neurotherapeutics.

## Data Availability

Not applicable.
